# Semantic Interoperability for IoT Platforms in Support of Decision Making: An Experiment on Early Wildfire Detection

**DOI:** 10.3390/s19030528

**Published:** 2019-01-27

**Authors:** Nikos Kalatzis, George Routis, Yiorgos Marinellis, Marios Avgeris, Ioanna Roussaki, Symeon Papavassiliou, Miltiades Anagnostou

**Affiliations:** Institute of Communication and Computer Systems, 15773 Athens, Greece; nikosk@cn.ntua.gr (N.K.); groutis@cn.ntua.gr (G.R.); gmar@image.ntua.gr (Y.M.); mavgeris@mail.ntua.gr (M.A.); papavass@mail.ntua.gr (S.P.); miltos@cn.ntua.gr (M.A.)

**Keywords:** data interoperability, Internet of Things, decision making, social media

## Abstract

One of the main obstacles towards the promotion of IoT adoption and innovation is data interoperability. Facilitating cross-domain interoperability is expected to be the core element for the realisation of the next generation of the IoT computing paradigm that is already taking shape under the name of Internet of Everything (IoE). In this article, an analysis of the current status on IoT semantic interoperability is presented that leads to the identification of a set of generic requirements that act as fundamental design principles for the specification of interoperability enabling solutions. In addition, an extension of NGSIv2 data model and API (de-facto) standards is proposed aiming to bridge the gap among IoT and social media and hence to integrate user communities with cyber-physical systems. These specifications have been utilised for the implementation of the IoT2Edge interoperability enabling mechanism which is evaluated within the context of a catastrophic wildfire incident that took place in Greece on July 2018. Weather data, social media activity, video recordings from the fire, sensor measurements and satellite data, linked to the location and the time of this fire incident have been collected, modeled in a uniform manner and fed to an early fire detection decision support system. The findings of the experiment certify that achieving minimum data interoperability with light-weight, plug-n-play mechanisms can be realised with significant benefits for our society.

## 1. Introduction

The Internet of Things (IoT) is reaching new levels of popularity and maturity with high penetration in various application domains directly influencing the societal and economical aspects of our lives. The concept of IoT, initially utilised as an umbrella term for a range of various emerging technologies, e.g., “embedded internet” and “pervasive computing”, is currently paving its own way being the key driver for digital transformation in several industries such as manufacturing, automotive, health, smart cities, smart farming, etc. IoT growth is explosive and there are already billions of connected smart objects, embedded systems, microcontrollers and sensors that have penetrated our world connecting home users, businesses, public facilities and enterprise systems with one another. However, there are various obstacles to further progress with the IoT vision and innovation, such as matters regarding security, privacy, user acceptance, data sharing, to name a few. One of the critical issues is that today’s IoT landscape consists of platforms and proprietary systems that are mainly isolated and act as “vertical silos”. These silos impede the creation of cross-domain, cross-platform and cross-organisational services due to their lack of interoperability and openness. As stated in [1], interaction among IoT systems can further improve produced economic value as most of the sensed data are not currently exploited to their full extend, while in some cases interoperability is a mandatory enabler for capturing the maximum potential worth. Among the technological and economical drawbacks caused by the lack of platform interoperability are the difficulties regarding integration of heterogeneous IoT devices into platforms, difficulties regarding the development of applications exploiting data from multiple IoT platforms, obstacles of IoT technology utilisation at a large-scale, increased costs and discouragement in adopting IoT technologies, and overall user dissatisfaction [2]. On the other hand, enabling interoperability among platforms provides many benefits including the bridging of vertical silos and the cooperation among platforms to offer advanced solutions to the end-users paving the way to new market opportunities. The cross-availability of services and data will allow new business opportunities to emerge from the ability to manage data from diverse sources to create innovative solutions allowing current service providers to reach new markets [1].

In computer systems, the term of interoperability is associated with “the ability of two or more systems or components to exchange data and use information” [3]. However, the dynamic nature and the complexity of information involved in cross-domain IoT applications require more advanced mechanisms for data modeling and management. To this end, the concepts of syntactic and semantic interoperability have been introduced. The concept of Syntactic Interoperability is associated with a set of formal data format specifications and the ability of systems to exchange information in order to communicate on a technical abstraction level [4]. Syntactic interoperability can be considered as a prerequisite to Semantic Interoperability [5], which refers to the ability of different applications and business entities to understand exchanged data in a similar way, implying a precise and unambiguous meaning of the exchanged information.

One of the first steps towards an interoperable IoT has been the approach known as the Web of Things (WoT) [6,7]. The WoT introduced a common stack for the IoT based on Web Services following the RESTful architecture. However, even when a homogeneous access was reached through Internet and Web protocols such as HTTP and CoAP, a common understanding was not yet acquired. For this purpose, the Semantic Web of Things (SWoT) was proposed for the integration of the Semantic Web on the WoT. Currently there are significant ongoing efforts for the definition of common semantics aiming to reach a consensus among the diverse data modeling approaches.

**Figure 1:**
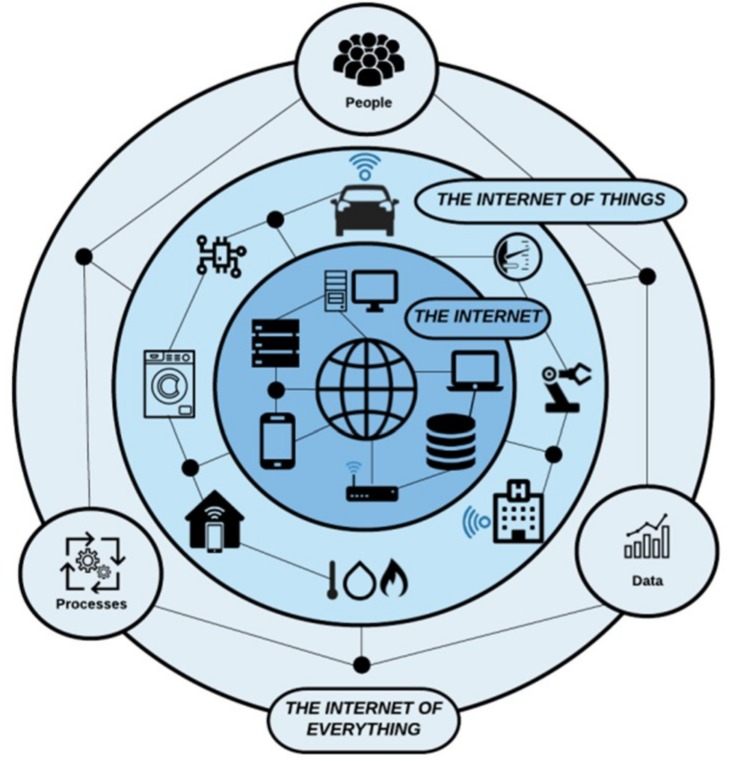
Cross-domain data interoperability is the key enabler for the evolution of the Internet of Things to the Internet of Everything.

Cross-domain interoperability is expected to be one of the main drivers for the realisation of the next state of IoT computing paradigm which is already getting shape under the name of Internet of Everything (IoE). As it is illustrated in Figure 1, the IoE aims to go beyond connecting just devices and sensors and to “bring together people, process, data, and things to make networked connections more relevant and valuable than ever before-turning information into actions that create new capabilities, richer experiences, and unprecedented economic opportunity for businesses, individuals, and countries” [8].

To this end, beyond the connectivity of physical devices and objects currently facilitated by the IoT, the IoE aims to define the appropriate mechanisms that will allow the seamless interconnection of people’s communities through the integration of technologies such as the social media services and crowd sensing paradigm with IoT data analytics and intelligent decision making. Of course, there are still a lot to be resolved and there are important issues that go beyond the technical obstacles. Issues that are affecting ethical and societal aspects as user acceptance is the core driving power to further establishment of new techs and services in everyday life. In fact, as indicated by Gartner’s top 10 IoT trends for 2019 and beyond [9], social, legal, ethical issues and user experience are the most intriguing among them. Successful deployment of an IoT solution demands that it’s not just technically effective, but also socially acceptable.

The work presented in this article analyses the current status and the significant work already conducted during the recent years in IoT interoperability efforts in order to identify a set of generic requirements that act as fundamental design principles for achieving a system-of-systems approach tailored to the IoT ecosystem’s needs. In addition, and most importantly this work adapts and extends (de-facto) data model and API standards for bridging the gap among IoT and social media and hence to integrate user communities with cyber-physical systems. Although there are already existing attempts towards this integration, these approaches are not considering the use of standards based data models and interfaces. Finally, the introduced data interoperability mechanisms are evaluated in the scope of a demanding and time critical use case that targets the early detection and monitoring of forest wildfires. It should be noted that the work presented in this article extends the interoperability mechanisms described in [10] that were initially applied and tested on Future Internet Research and Experimentation (FIRE+) testbeds.

This article is organised as follows: Section 2 elaborates on the related work, focusing on three domains: interoperability in IoT, exploitation of social networks as sensing tools and existing IoT solutions in support of emergency situations. Section 3 introduces the data interoperability mechanism proposed for IoT systems and Section 4 presents the respective system architecture developed. Section 5 elaborates on the setting and rationale of the experiments designed to evaluate the proposed framework, using among others a real wildfire incident that occurred in July 2018 in Greece that completely destroyed a semi inhabited forest area near Athens and caused the death of 100 people. Section 6 presents the experiment executions and the respective evaluation findings and Section 7 concludes the paper and discusses directions for extensions of the presented work.

## 2. Related Work

### 2.1. Interoperability in IoT

In the systematic literature review presented in [11,12] there is a clear indication that the topic of interoperability of IoT systems is gaining an increased attention from the research community especially during the recent years. As it is analysed in the review the dominant approach for facilitating data interoperability in IoT is the adaptation of Semantic Web languages, tools and technologies utilised for the description and discovery of things and services, the composition of services, but also for applying reasoning algorithms over IoT resources. In a similar manner, the W3C organisation has recently launched the Web of Things Working Group (https://www.w3.org/WoT/) in support of the definition of initial semantic interoperability standards, aiming to counter the fragmentation of the IoT and reduce the overall costs of development. W3C aims to achieve this by enabling expressions of the semantics of things and the domain constraints associated with them, building upon W3C’s extensive work on Semantic Sensor Network (SSN) ontology (https://www.w3.org/TR/vocab-ssn/), RDF and Linked Data technologies.

Another well-established approach for IoT interoperability at a platform level is the Next Generation Service Interface (NGSI) [13] which is currently a de-facto standard that has been released by Open Mobile Alliance (OMA) and it supports the receiving, processing, contextualising, and publishing of IoT data. The NGSI API and the associated NGSI context model has been adopted by key organisations in the IoT standardisation efforts such as ETSI and AIOTI WG3 while a RESTful binding of OMA NGSI-9/10 [14] has been defined by the FIWARE [15] initiative through the implementation of the Orion Context Broker. In the smart cities application domain, an open and agile smart cities initiative [16] has been signed by 31 cities from countries such as Finland, Denmark, Belgium, Portugal, Italy, Spain, and Brazil, that adopted the NGSI open standard in their smart city platforms.

The importance of interoperability in IoT ecosystems is underlined, among others, by the fact that EU has invested considerable efforts expressed both as financial and administrative support in pushing forward the cross-domain, cross-platform IoT paradigm. On 2015 a number of research and innovation projects where funded with 50M euros aiming to deliver architectural concepts for semantic interoperability for “Platforms for Connected Smart Objects”, aiming to cover multiple use cases whilst responding to specific requirements in terms of security, dependability, cognition and prioritised event processing. As these research projects are currently reaching their finalisation interesting outcomes are presented through an EU IoT platform development initiative called IoT-EPI (https://iot-epi.eu/) and the respective book entitled “Advancing IoT Platforms Interoperability Book” [2]. 

In more details the AGILE project [17] designed and developed an information gateway focusing on the physical, network communication, processing, storage and application layers of the IoT stack. In addition, the designed software modules provide functions that facilitate device management, area and sensor communication networks and distributed storage. The project considers all the modules needed to provide a robust security management solution. 

The bIoTope project [18] aims to address the interoperability problem for the various layers of the IoT stack by providing a set of recommendations but also the specification of an architecture along with the respective implemented solutions based on standardised open APIs. The architecture facilitates the use of open standards and use case exemplar implementations that support stakeholders to create new IoT systems and services and to harness available information using a Systems-of-Systems approach. Finally, the interoperability solutions are validated in a cross-domain environment. 

The BIG IoT project [19] aims to develop a generic API based interoperability solution targeting mainly the upper layers of the IoT architecture. The project defines mechanisms that enable platforms to register with a public directory in order to be discoverable but also defines the necessary semantics facilitating uniform data exchange. In general, it develops generic mechanisms that are addressing security management, internal and external service integration. The developed solutions initially deployed on 8 IoT platforms maintained by the project’s consortium partners. As a next step, additional platforms are expected to be integrated via the project’s community building process. 

The INTER-IoT [20] project targets six layers of the IoT architecture through an open cross-layer framework, an associated methodology and tools to enable voluntary interoperability among heterogeneous IoT platform. The developed modules are covering the QoS and device management, service integration, external system services, storage and virtualisation. In addition, the developed solutions aim to provide a full security management suite.

The symbIoTe [21] project aims to tackle cross platform interoperability by providing an abstraction layer enabling a unified view on various IoT platforms and sensing/actuating resources. Applications can use symbIoTe Core Services implementing a semantic IoT engine to find adequate resources offered by symbIoTe-enabled platforms and subsequently access platform’s virtual resources directly for data acquisition and actuation. In addition, the project focuses on seven layers of the IoT architecture from physical to application layer and proposes a full security management suite. 

The VICINITY [22] project provides interoperability mechanisms targeting the five-upper layer of the IoT architecture through the specification of a platform that provides “interoperability as a service”. The work considers the service integration, business logic, virtualisation, storage, APIs, tools, external system services, applications, data analytics and cloud services.

In a similar manner, on 2016, the IoT European Large Scale Pilots (LSPs) Programme [23] was launched aiming to foster the deployment of IoT solutions in Europe through integration of IoT technologies across the value chain, demonstration of multiple IoT applications at scale and in a usage context, and as close as possible to operational conditions. The programme was funded with 120 M euros while the targeted application domains included Smart Cities, Wearables for Smart Ecosystems, Smart Farming and Food Security, Autonomous Vehicles and Smart Living Environments for Aging Well. Within this initiative, the utilisation of open technologies and architectures that enable cross platform interoperability is among the main priorities. Within the scope of these projects significant effort is conducted toward the analysis of the current status of deployed IoT systems and potential interoperability solutions. These research projects are more focused on defining guidelines and principles that interoperability solutions should follow towards the realisation of a system-of-systems approach rather than developing centralised middlewares and platforms.

The system-of-systems approach is also the design principle for the Arrowhead Framework [24] which aims to support the development, deployment and operation of interconnected, cooperative systems based on a Service Oriented Architecture philosophy. The framework introduces an “Interoperability Layer” which encapsulates the appropriate data translation systems and services facilitating the interoperable interaction among heterogeneous IoT systems. In addition, the framework identifies the lack of understanding between various development groups as one of the core problem for developing highly interoperable systems hence adequate development and service documentation are provided. 

Besides the various efforts originating by EU based initiatives, the importance of IoT interoperability has been identified by numerous standardisation organisations such as IEEE, IETF, W3C, OMA, AllSeen Alliance, OCF, NIST, CableLabs, ZigBee, and ETSI. These leading bodies joined their forces aiming for consensus building with regards to interoperability of Things as a mean to drive development and standardisation in IoT. A first outcome produced by this initiative is the joint white paper entitled “Semantic Interoperability for the Web of Things” [25] which is co-authored by an informal group of experts from a broad range of backgrounds all of whom are active in standards groups, consortia and/or alliances in the Internet of Things (IoT) space. This white paper aims to create a mindshare on approaches to semantic interoperability and to actively encourage consensus building through technical recommendations.

Based on the aforementioned approaches it is obvious that there are significant ongoing efforts in support of interoperability for IoT services. Most these approaches are targeting the full stack of IoT architecture from the lower layers (e.g., networking and communication protocols) to the upper layers (e.g., IoT based applications) aiming to produce integrated solutions addressing the IoT interoperability in a holistic way. In addition, the various EU research projects produce a set of guidelines and recommendations mainly targeting the design of future interoperable IoT systems and not so much existing operational proprietary platforms. Toward this scope the proposed approaches tend to be rather complicated aiming to address all together in one combined solution the issues raised. Thus, the integration effort tends to be hard raising significant administrative issues. It should be noted, that the BiG-IoT project differs as it focuses more on the higher layers of the IoT stack and has among its priorities the integration of existing operational platforms. Another useful result is that the NGSI API and data model can be considered as the one of the dominant approaches for achieving openness and interoperability mainly in the areas of IoT and smart cities. However, none of the analysed initiatives foresees the specification of technical means for integrating existing IoT platforms with information sources related with peoples’ communities e.g., through the integration with social media services following open and dominant data modeling standards such as NGSI.

### 2.2. Social Networks as a New Sensing Paradigm

In the context of the Participatory Web or simply Web 2.0, social media provide an effective, sophisticated and powerful way to gather preferences and activities of groups of the population. For example, data that are produced on the social media services can generate a complex and adequate knowledge on a plethora of fields of application, such as economy (stock market analysis [26] and private consumption prediction [27]), politics (opinion polls [28] and predictions of political elections [29]), sports (predict football game results [30]), tourism (places to be visited by observing the most frequently attended places in a given location [31]), demographics (identifying gender and age of selected user groups [32]) and infotainment [33]. Approaches for exploiting social media data are already mature enough, going beyond research prototypes, to mature robust data analytics software products such as Sysomos, Keyhole, Agorapulse, and the Twitris platform.

In this context and evaluating the potential for integrating Social Media with IoT, recent innovations in data sensing, collection, storage and analysis have enabled the realisation of the so-called Social Sensing paradigm which is based on the idea that communities or groups of people can provide information data similar to those obtainable from a single sensor. In the case of an event or a disaster, the collection of this additional information can support to the grater situational awareness and to alert affected parties promptly or verify information obtained through other channels. In this direction, the evaluation of the impact of the data collected from the social media in relation to extreme weather incidents [34], earthquake events detection [35], and the estimation of diseases spread such as influenza [36] and malaria [37] has been recently studied extensively. These early results suggest that, although a robust methodology has to be defined and validated, this information can provide a promising approach for detecting and mapping environmental hazards and climate-related impacts. In the particular domain of wildfire detection, authors in [38] have studied the extent to which social media users in the proximity of a fire incident, can provide valuable information and testimony about the current situation and support the timely and accurate fire detection. The approach’s potential has also been confirmed by European Commission’s JRC initiative named Digital Earth Nervous System [39]. Furthermore, in [40], a review on the utilisation of social media as a powerful crowd-sensing tool for situation awareness and data diffusion in assistance of forest wildfires detection has been presented. There, the wildfire risk management systems and the social media methodologies followed, crowd-sourcing applications developed and social media frameworks deployed for disaster management were categorised, while a general architecture of a wildfire social sensor management platform and a sensing process based on social media data management were proposed. Regarding our approach on leveraging social media, the following two studies are the most relevant and dominant. A Twitter-based spatiotemporal content analysis for wildfires was provided by Wang et al. in [41], where the authors utilised the Kernel Density Estimation (KDE) method to analyze the possible spatial patterns of the tweets referring to the wildfires and they combined it with the temporal evolution of the tweets and a term frequency analysis to validate the ability of social media to characterise an emergency over time and space. Following a slightly different approach, Twitcident [42] was a web-based system connected to emergency broadcasting services that automatically searched, filtered and classified emergency situations while analytical tools and users were allowed to make customised searches for specific events, including wildfires.

As it was the case with the presented IoT interoperability approaches, the aforementioned social media analytics approaches are not attempting to integrate with IoT services and are not considering the challenge of semantic and syntactic interoperability. To this end, the presented approaches are mainly focusing on performing accurate and robust data analytics mechanisms while the mediation of the achieved result through open standards is neglected. Hence, fusion with additional valuable information sources is not feasible limiting the potential for advanced, real-time decision making.

### 2.3. IoT in Support of Emergency Management

Around the globe, many wild-fires take place throughout the year and this issue has been attracting significant research interest resulting to a huge amount of very well studied IoT-based solutions available for testing or even ready for use to resolve this problem. Some of the more interesting and complete state of the art reviews for such systems are provided by [43,44].

Within the scope of the work presented in this article we focus in lightweight, low cost hardware approaches. To this end, in [45], the authors describe a method for identifying a forest fire with the use of Arduino UNO, Raspberry Pi, where each Arduino contains a specific address. Every Arduino gets the analog values from its sensors and compares them to a threshold value. If the threshold value is exceeded, an alert notification is sent to the user. Every Raspberry Pi is connected to four sensors (rain sensor, smoke sensor, fire sensor, temperature sensor) and sends the signals through a GSM modem. The GSM modem, however is high energy consuming, affecting the battery lifetime. In [46], it is proposed a solution, based on Arduino, where each module senses temperature (−40 °C to +85 °C), humidity (0–100%), atmospheric pressure (30–110 kPa) and pollutant gases: CO (30–1000 ppm), CO_2_ (350–10,000 ppm). It communicates with a web service using 4G modem. When data are available, the information is sent to the Web service, which is responsible for managing the resources. Web service is capable of storing measurements and manages their visualisation by using graphs. There is also use of an Android mobile application with the aim of representing locations of IoT devices, measures and averages for each variable monitored (with the use of graphs) which users can easily access. It is positive that this solution uses graph representation and easy access through mobile application, but again 4G modems are energy harvesting solution and when batteries are used, it affects the device’s lifetime. In Ref. [47], there is a description of a construction that is based on Arduino Uno. This module is connected with an Ethernet module, LCD, fire sensor, smoke sensor (MQ-6), buzzer and an ESP8266 module (through SPI port). The sensors measure humidity, temperature and smoke from the environment, and if a set threshold is exceeded, the buzzer is activated, a message is sent to the forest department so that satellite tool can take images and the forest department to handle the situation. It is positive that ESP8266 module―which is wireless modem―is used, so that the device does not depend on cables. These types of WI-FI modules that operate at 2.4 GHz, have a good bitrate (72 Mbps) but they cannot reach more than 100 m in open space. The LCD can inform the user for the sensors’ values, but in the case of using many devices in an open area, it is useless to have on each device an LCD module, because they can quickly inform the center in case of emergency situation through the WI-FI module. In [48], there is a description of a more robust model for using IoT technology for fire monitoring system which consists of three parts. The first layer is about data analysis. It transforms the temperature, humidity and image to data and sends it through the serial port. The second layer is used in order to process the data that have been obtained, using different algorithms to detect warning of fire and alarm warning. These algorithms are: threshold algorithm, comparison algorithm, fuzzy logic algorithm and algorithm which is based on Dempster-Shaffer Theory. Fuzzy logic algorithm, for example, is based on information fusion. Multi sensors or multi sources are analyzed or synthesized, considering certain criteria in order to complete data processing for decision making and task estimating. This is realised by fusing fire parameters of multi sensor with the aim to determine fire occurring (or not) based on different kinds of fire parameters. The third layer concentrates on the interaction layer, where it displays the data collected from the sensors. The data curve is analyzed with the use of previously collected data while web access is provided to users in order to get information in real time.

As it is indicated from this review, micro-environmental sensing solutions based on low cost hardware platforms such as Raspberry and Arduino are mature enough and able to produce significant results towards the early detection of forest fires. In addition, automatic recognition of fire related incidents through image and video analytics is a solution that is already deployed in operational environments. However, data interoperability mechanisms capable to integrate IoT measurements with social activity related data are still in an early research stage.

## 3. IoT and Standards-Based Data Interoperability Mechanisms

### 3.1. Current Status on Data Interoperability in IoT

There are various reasons for not having reached yet a consensus on IoT related protocols, data models and APIs towards the establishment of an interoperable ecosystem. A common requirement for computer services and applications that are built for IoT systems is to be able to operate with limited storage, computational and energy resources. At least, this is the case for the devices deployed and operating at the edge of the IoT networks such as sensors, actuators, gateways and other small devices. In addition, in many cases existing IoT deployments are aiming to serve highly demanding domains that need to operate 24/7 such as fleet/asset management, manufacturing, smart metering, advanced building technologies and smart farming systems. Scarcity of resources combined with security and system administration related requirements are forcing engineers to apply custom, simple and practical solutions, tailored to the needs of each application domain, having as first priority the sound operation of the services. Introducing data interoperability mechanisms to existing operational IoT environments raises questions about the overall system performance, integration efforts required, introduced cost and return of investment thus making the overall effort not an easy task and system administrators reluctant to proceed.

Another obstacle towards achieving interoperability is the lack of common standards. The current status in standardisation process is that in order a new approach to be accepted as a standard it is necessary to be already adopted and utilised by a large number of entities active on the targeted domain (e.g., companies, working groups, research communities, and other initiatives). On the other hand, in order to reach a critical mass of entities that will widely accept the introduced solution requires the definition of common practices, protocols, models, and practical examples. As it is obvious this process can drive to a “chicken and egg situation” and never reach the required consensus. A solution to this situation that seems to be adapted successfully in various application domains is to target the realisation of a “minimum interoperability level” [49] among systems. Having established a minimum consensus, it is then feasible to reach a production operational level from an early stage and then start building on top of these more advanced solutions.

**Figure 2:**
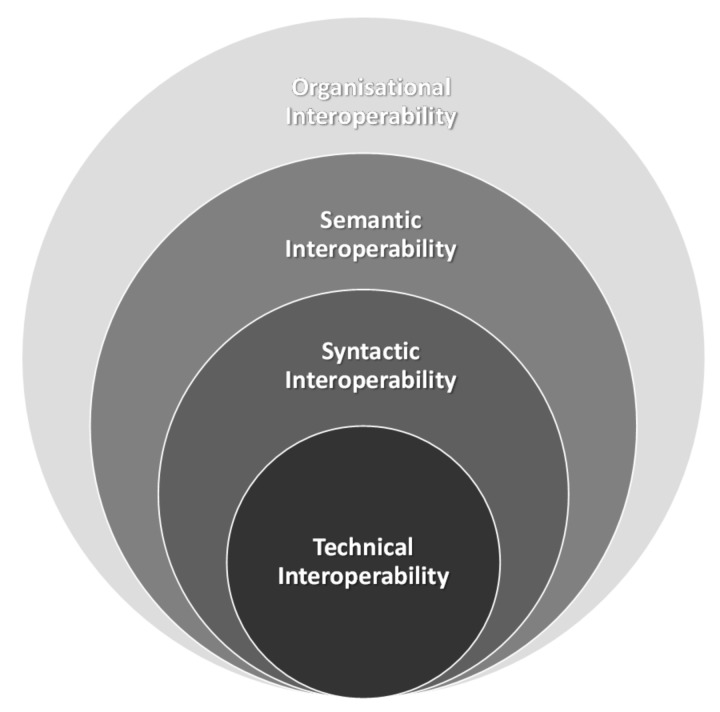
The dimensions of interoperability [3].

There have been various efforts for defining a methodology in order to address the interoperability challenge in IoT. In the related literature, there exist various classifications of the interoperability aspects, which are also called levels of interoperability. For example, the European Interoperability Framework designed “to support the delivery of pan-European eGovernment services to citizens and enterprises” [50] defines the following three levels: technical, semantic and organisational interoperability. In the report presented in [3] and also illustrated in Figure 2 an IoT-specific classification split in four levels is presented:

- Technical Interoperability: usually associated with communication protocols and the infrastructure needed for those protocols to operate.
- Syntactic Interoperability: usually associated with data formats and encodings, e.g., XML, JSON and RDF.
- Semantic Interoperability: associated with a common understanding of the underlying meaning of the exchanged content (information).
- Organisational Interoperability: associated with the ability of organisations to effectively communicate and transfer information even across different information systems, infrastructures or geographic regions and cultures.

In the work presented in [51] for the needs of IoF2020 Large Scale Pilot, a more practical approach is followed and various points are identified where potential interoperability solutions can be applied while the various points are associated with existing standards. Based on this approach along with the ITU-T Y.2060 IoT architecture described in [52] we present in Table 1 the coupling of the defined IoT layers with the respective dominant associated standards.

**Figure 3:**
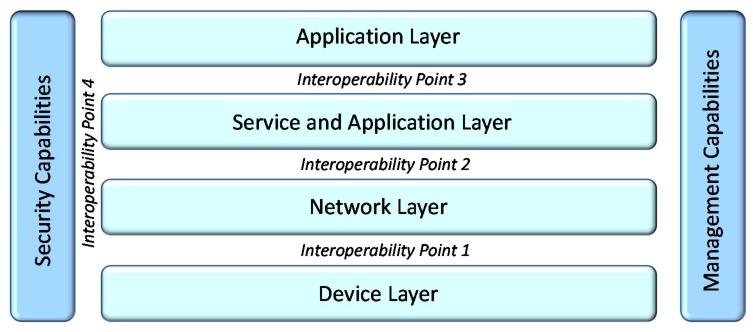
IoT reference model (ITU-T Y.2060) and the points where interoperability solutions can be applied.

A special viewpoint of the schematic representation of the ITU-T Y.2060 IoT Layered architecture along with the interoperability points is illustrated in Figure 3.

In the scope of the work presented in this article we focus on cross-domain and cross-platform interoperability. The data interoperability mechanisms to be detailed described in the following sections aims to enable cross-domain data interoperability enabling information fusion from diverse systems such as social media services with IoT environmental sensing platforms. Another design decision of the proposed solution is that aims to achieve an optimal balance between deployed interoperability mechanisms and undisturbed operational and administrative status of existing systems, hence the proposed solution is designed to operate on top of Service Application Layer targeting Interoperability Point 3. 

### 3.2. The NGSI and NGSI-LD Information Models

As it was presented in Section 2.1, there are various efforts and initiatives towards standardisation in support of data interoperability for IoT ecosystems in operational environments where the NGSI API and data model is considered as a robust candidate solution. With regards to IoT interoperability on a platform level, ETSI created an Industry Specification Group on cross-sector Context Information Management (ISG CIM) for IoT-enabled Smart Cities and also other verticals including Smart Industry and Smart Agriculture. The group focuses on developing specifications for a common API, Data Publication platforms and standard Data Models, in order to achieve and improve cross-sector interoperability for smart applications, with Next Generation Services Interface (NGSI) API as its starting point. Moreover, the AIOTI WG3 [52] also proposes the utilisation of OMA NGSI API for the interconnection of IoT platforms and for integrating IoT data with other data pools. The EU based Large Scale Pilot projects targeting the Smart Cities (Synchronicity [53]) and Smart Agri-food (IoF2020 [54]) domains are also adopting the NGSI as a candidate information model and API for further building data interoperability mechanisms. Based on all these it can be safely assumed that the NGSI API and data model [55] has gained the required consensus in order to be considered as a commonly agreed tool for realising data interoperability in IoT.

**Figure 4:**
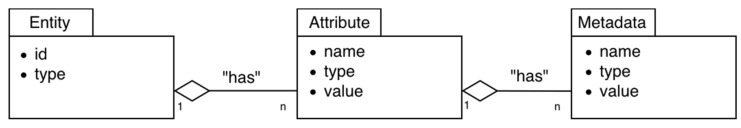
The OMA NGSI meta-model [55].

The NGSI context information model is capable to support semantic metadata along with already defined data models for various use-case areas (environmental monitoring, transportation, weather, points of interests, devices, alarms, civic issue tracking, etc.). The core element in the NGSI context interfaces is the concept of entity as it had been used in the context definition by Anind Dey [56], which is relatively popular in the context-awareness community: “Context is any information that can be used to characterise the situation of an entity”. As shown in Figure 4, the Context Entity (e.g., physical object, person, place, thing) apart from id and type fields (that define the ID and type of the entity), also contains a payload of a set of attributes. Each attribute contains a name, a type and a value and possibly a number of meta-data elements but it is also possible to utilise more complex structures for modelling attributes. Given that the NGSI Entity/Attribute data model doesn’t specify any strict semantics it is still possible for inconsistences among IoT platforms utilising this data model to arise. For this reason, the FIWARE initiative [57] along with the GSM Association [58] have defined a set of domain specific vocabularies for the NGSI data model targeting various application domains such as: Alerts, Building, Civic Issue Tracking, Device, Environment, Smart Cities Key Performance Indicator, Parking, Parks & Gardens, Points of Interest, Street Lighting, Transportation, Weather. FIWARE and GSMA have also defined a process and best practices for reusing and extending these data models for areas that are not currently covered.

As already stated the ETSI organisation is currently in a process to define a new standard for the IoT domain having as starting point the existing OMA NGSI-9/10 specification incorporating the latest advances from Linked Data. For this reason, this new standard is also known as NGSI-LD. In a similar manner, the NGSI API implementation named Orion Context Broker is evolving as an open source reference implementation of the new ETSI NGSI-LD API specifications. Although the official release of the standard is expected on March 2019 a preliminary specification [59] has already been publicly available in order to elicit comment and critique from the ICT community. These comments will be considered for future modification and for the overall improvement of the later final API specification. The preliminary specification formally describes the Context Information Management API which allows users to provide, consume and subscribe to context information in multiple scenarios and also enables close to real-time access to information coming from many different sources that are not limited only to IoT data sources. ETSI aims the new standard to be considered as a cornerstone for the development of Smart Cities, Smart Agrifood and Smart Industry applications.

**Figure 5:**
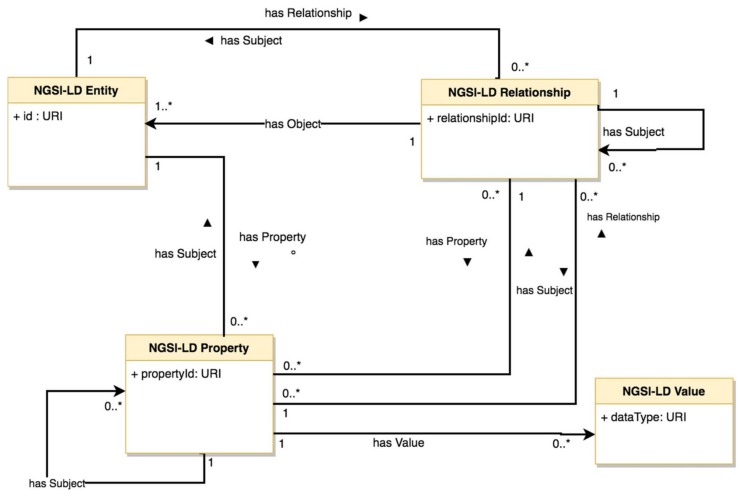
UML representation of ETSI ISG CIM (NGSI-LD) information model [59].

In Figure 5, the UML representation of ETSI ISG CIM information model is illustrated. The main constructs are the NGSI-LD Entity, NGSI-LD Property and NGSI-LD Relationship. Instances of NGSI-LD Entities can be the subject of NGSI-LD Properties or NGSI-LD Relationships. Compared to the traditional NGSI data model, all the Entity, Property, Association and Value data types are possible to be associated with unique URIs corresponding to well-established semantic identifiers. Thus, it is possible to utilise RDF syntax expressed in JSON format. The NGSI-LD Relationships allow for establishing associations between instances using linked data. In practice, they are an NGSI attribute, but with a special value which happens to be a URI which points to another entity. 

### 3.3. Design Requirements for Data Interoperability Mechanisms

Having analysed the current status regarding interoperability in IoT, a set of core requirements are identified that aim to act as fundamental design principles for future data interoperability mechanisms. The set of these requirements aim on the development of interoperability mechanisms able to be deployed on top of existing operational systems towards the realisation a system-of-systems approach. In future, there might be the case that IoT deployments will confront to standards by their very initial design and hence system interoperability will be an inherent feature. However, as it is already analysed the current IoT ecosystem is far from such a realisation, hence the development of interoperability enablers for existing IoT systems is considered of high priority:

- Standardised semantics: This is an obvious requirement but still the most significant. The information model to be selected for facilitating data interoperability should comply with standards and demonstrate all these features that are required for operating in the targeted application domain. The fact that in some cases there are already multiple standardised data models possible to be applied on the same application domain complicates things even more introducing the need for cross-standard interoperability mechanisms.
- Seamless integration: The interoperability mechanisms should not affect the existing operation of the IoT deployment or to affect it as minimal as possible. As it is already stated real world, operational deployments tend to be complicated including various customised engineering approaches. In addition, it is not realistic to expect custom proprietary solutions to change their mode of operation in order to adapt with changes imposed towards the realisation of interoperability. Hence it is the interoperability enabling solution that needs to be versatile and easy to adapt to the underlying system. Hence a Plug-n-Play and light-weight (as possible) software design is necessary for seamless integration with existing systems.
- Information completeness: Data interoperability often imposes the use of data translation services. The interoperable data model and the data conversion mechanism should be able to support the available proprietary/customised information elements as much as possible. Although it might not be necessary to expose the full set of information entities as some of them might only useful for internal use the interoperability mechanisms should have the capacity of modelling and managing the full set of data.
- Data volume optimisation: The use of semantics as the main tool to achieve data interoperability has as a consequence the overall increase of the data volume. Operational IoT systems tend to minimise the overall data volumes using simplified formats modelled for example in CSV and JSON. Translating these data to semantic expressions based on XML, RDF, OWL, etc. introduces a significant overhead that in many cases is unrealistic to be handled by resource constrained devices and scarce network bandwidths. Hence it is necessary to reach a balance between interoperable semantics and data volume increase.
- Control of information flow: The administrative entity that owns or manages the IoT data should have the means to control which information elements are leaving their cyber-premises and how it will be utilised. Hence, the interoperability enabling mechanisms need to be easy to use and clear administration and configuration operations are necessary to be available in order to be widely accepted.
- Security and Privacy protection of individuals: A common challenge that needs to be addressed in the years to come is to keep a balance between monitoring of every-day activities for various application domains without on the same time violating human rights such as the right of privacy protection of individuals. Advances on sensing technologies and data collection mechanisms make feasible the deployment of vast sensor networks that can potentially become intrusive and violate established regulations (e.g., GDPR). Edge computing paradigm assists in keeping the processing at the edge of the network thus avoiding the indiscriminate transmission and storage of sensitive information, such as image and video recordings.
- Reuse: During the recent years, there is a significant effort invested on IoT interoperability solutions. However, there is a lack of coordination in these efforts which causes many initiatives to evolve in parallel and even having overlapping standards for the same application domain. In fact, it might be possible in the future to have the need for cross-standard interoperability solutions. One solution for this issue is the reuse of existing informational models and implementations, as much as possible, instead of reinventing the wheel with new approaches. In addition, tools and tutorials are necessary in order to ease developer tasks, running pre-made scenarios, along with solutions to reduce standardised technologies learning curve.

## 4. System Architecture 

### 4.1. The IoT2Edge Interoperability Mechanism 

Based on the requirements defined in the previous section, the IoT2Edge data interoperability mechanism is specified. The IoT2Edge follows an agent-server architecture, where the respective modules are called IoT2Edge-Agent and IoT2Edge-Server and provide syntactic and semantic interoperability mechanisms targeting existing operational information platforms. The IoT2Edge-Agent aims to be as lightweight as possible and easily deployed on top of existing IoT platforms as a plug-n-play module targeting interoperability level 3 as this is defined in Table 1. However, the IoT2Edge-Agent is not only targeting IoT platforms, but aims to be able to connect with information platforms that provide publicly available and well specified APIs. In general, the IoT2Edge-Agent act as an interoperability enabler by translating data derived from the underlying platform in the NGSIv2 context information model. The NGSIv2 model have been selected as an appropriate common “language” as it is already being widely accepted in various application domains and demonstrates a set of characteristics that make it capable to bridge the IoT with other domains as it is argued in Section 3.2. 

For the design of the IoT2Edge-Agent the “microservices” architectural approach has been adopted. IoT2Edge-Agent components are bundled as distinct small services where each one is running as a separate process communicating through simple mechanisms. This architectural approach is followed in order avoid one monolithic service and to have a number of microservices that are able to evolve relatively independently from each other and to communicate in a safe, secure and efficient manner. Inter-process communication is facilitated either synchronous or asynchronous (e.g., message broker). 

**Figure 6:**
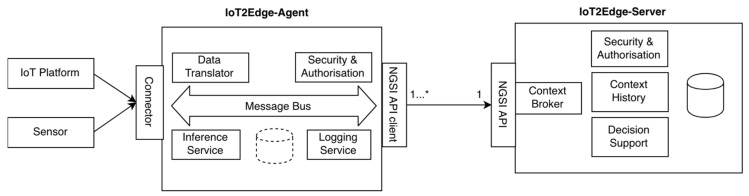
Functional view of the proposed IoT2Edge Agent-Server architecture.

A short description summarising the functionality of each microservice is presented here-after while a functional view is illustrated in Figure 6:

Connector: This module utilises the API of the underlying information platform in order to capture provided data streams that will be fed to the Data Translator. Appropriate security and authentication mechanisms are applied according to the policies imposed by the data providing platform in order to grand access to data. In addition, the Connector implements the dictated policies, if available, with regards to allowed API utilisation, limits of querying rates, etc.

Data Translator: The Data Translator uses as input the streams of data readings (e.g., sensor measurements) provided by the Connector component, which are derived from the various underlying platforms and translates these according to the NGSIv2 data model. The translator is tailored to each information platform’s data model specifications, while in general the IoT2Edge-Agent reuses existing NGSIv2 based semantics wherever possible (e.g., for environmental monitoring, for device representation, for description of events/alert, etc.). In some cases, it is not feasible to perform a direct conceptual mapping of the sensed data type with an information element already specified by the NGSIv2 data model. In order to address such situations a set of directions is provided in order to extend NGSIv2 information model with new concepts. More details on this process are described in the following Section 4.2.

*NGSI API* client: This module facilitates the communication with the IoT2Edge-Server utilising the NGSIv2 API. Essentially, it provides the necessary REST-API calls with the appropriately crafted JSON data objects in order data to be communicated with the Context Broker deployed at the IoT2Edge-Server. It also enforces the dictated security and authentication policies e.g., using the appropriate credential and authentication methods in order to expose the data.

Security and Authorisation: It ensures authentication, authorisation and confidentiality of disclosed data. It should be noted, that IoT2Edge-Agent follows a privacy protection by design approach for IoT platforms as it is deployed at the cyber-premises controlled by the same administrative entity that also manages the platform. This approach allows the full regulation of the information flow from the Connector to the Data Translator and the final provision through the NGSI API client.

Inference service: Infers high level information and assigns semantic labels based on the processing of low level raw data, a process that is complementary to the translation task. The logic of the inference engine can be customised according to the specificities of the respective application domain and the semantic technologies utilised in order to provide common means for describing domain knowledge. As it will be further analysed in the following section this step is crucial for identifying the appropriate annotations for characterising situations based on sensor readings. It might be the case that it is necessary to maintain more advanced classification engines and decision rules thus a lightweight storage is foreseen as an optional module of the IoT2Edge-Agent.

Logging Service: This module is not considered to be a core component for the overall functionality of the Agent. However, it is necessary within the scope of this work as it monitors the usage of various system resources (e.g., CPU/storage/bandwidth utilisation, RTTs, etc.) during the execution of the experiments. It is responsible for coordinating the collection of the necessary datasets to be later used for the scientific evaluation of the proposed approaches. The IoT2Edge-Server handles storage of aggregated data received from various Agents, which are uniformly modelled based on the NGSIv2 data model. Communication with the Agents and other consumers is established via the NGSI API. The IoT2Edge Cloud Service includes the components below:

Context Broker: This module provides the necessary means for persisting data received from multiple IoT2Edge-Agents but also for maintaining higher level information inferred through from data inference processes. As the “reuse” of existing implementations is considered as a design principle (Section 3.3) the Orion Context Broker (OCB) is utilised along with an instance of Mongo DB. As it is already stated the OCB is a RESTful binding of OMA NGSI-9/10 specified as a Generic Enabler (GE) by FIWARE. The OCB supports synchronous & asynchronous (publish/subscribe) data retrieval and a simplified syntax to retrieve entities which match a set of conditions.

Context History: As OCB mainly stores current contextual information it is necessary to provide the means for persisting histories and logs of sensed data. For this reason, the reuse of the FIWARE GE called Cygnus [60] is employed. Cygnus is a connector in charge of persisting certain sources of data third-party storages, creating a historical view of such data. Internally, Cygnus is based on Apache Flume, a technology addressing the design and execution of data collection and persistence agents. An agent is basically composed of a listener or source in charge of receiving the data, a channel where the source puts the data once it has been transformed into a Flume event, and a sink, which takes Flume events from the channel in order to persist the data within its body into a third-party storage. Cygnus is designed to run a specific Flume agent per source of data. It is expected the Mongo DB repository to be utilised for persisting historic logs.

Security & Authorisation: This component provides the necessary mechanisms for ensuring that access to data is granted only to authorised entities and to the right level. Given that the FIWARE OCB and Cygnus are the core data-management components the respective security mechanisms are also based on the combination of FIWARE GEs such as Identity Management Keyrock and Authorization PDP—AuthZForce [61]. In addition, the appropriate mechanisms (e.g., data anonymisation, aggregation functions) will be provided in order to ensure compliance with respect to data-privacy and data protection rules as these are also indicated by GSMA IoT Security Guidelines [62].

Decision Making: Supports intelligent decision making based on semantics reasoning on collected data, e.g., evaluating how critical a situation is based on sensor readings. Depending on the application domain different decision algorithms can be implemented. An extensive analysis of the Decision Algorithm will follow tailored to the requirements of the conducted experiment.

### 4.2. The IoT2Edge Translation Process 

In this section the translation process realised by the “Data Translator” component of the IoT2Edge-Agent is elaborated. The conversion of a custom data structure to a standardised form is the key step towards the realisation of data interoperability. The “Data Translator” operation is based on the GSMA’s [58] directions and semantics vocabulary described in “IoT Big Data Harmonised Data Model” that have been defined in relation to the data context model NGSIv2 and its successor NGSI-LD. In addition, the set of requirements defined in Section 3.3 are also considered. The GSMA’s directions foresee the option of creating new vocabularies or extending existing ones with additional attributes in case concepts from the targeted use case are not covered. However, the introduction of new attribute types should be avoided as it can cause various inconsistencies and endanger the desired utilisation of common semantics. 

The translation process is essentially mapping sensor readings and other data elements to the targeted common model. In order to illustrate this process sensor readings from the Mobile Environmental Sensing Platform (MESP) are utilised. As it will be detailed described in the following section the MESP is a prototype platform consisting of various sensors capable to measure air temperature, soil temperature, air humidity, CO_2_, fire flames presence, etc. The MESP platform follows a minimalistic approach on modelling sensed data aiming to reduce the overall volume of transmitted and stored data. As it was analysed in the previous sections this is a standard approach for IoT proprietary systems that utilise resource constrain devices. A sample of the MESP recorder sensor measurements is the following:

recordID;nodeID;gps_lat;gps_lng;timestmp;humidity;flame;tempAir;tempSoil;airCO_2_;fireImgAlert;
050618161710;1234;37.979839;23.783744; 1528208228;6.00;1023;26.00; 26.44; 29; medium
050618161711;1234;37.979839;23.783744; 1528208230;6.00;1023;26.00; 26.44; 29; medium
050618161712;1234;37.979839;23.783744; 1528208233;6.00;1023;26.00; 26.44; 29; medium

**Figure 7:**
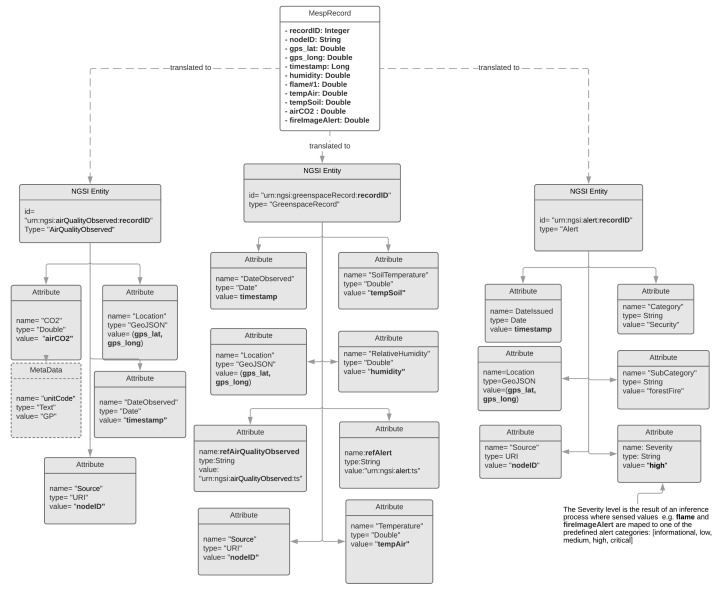
Data object model demonstrating the MESP record translation process to NGSI.

**Figure 8:**
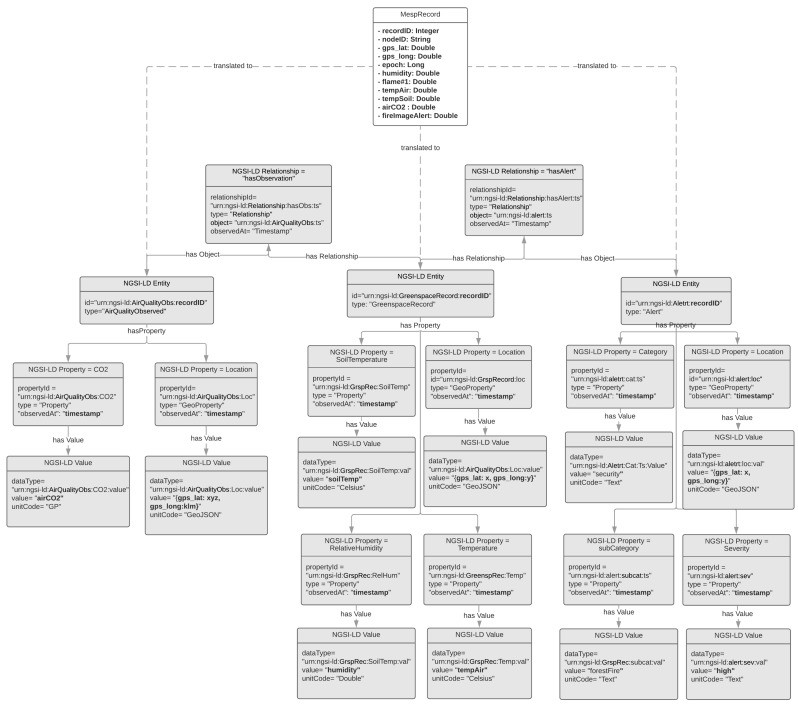
Data object model demonstrating the MESP record translation process to NGSI-LD.

The IoT2Edge-Agent has been deployed on MESP while the “Connector” is retrieving the respective measurements and forwards them to the “Data Translator”. The “Data Translator” logic on mapping MESP measurements to NGSI information entities is depicted through the data object diagram in Figure 7. In a similar manner, the same process but targeting the NGSI-LD information model is illustrated in Figure 8. In order to make easier the tracking of information the NGSI and NGSI-LD attributes derived from MESP-Record are illustrated with bold fonts. The approach that is followed for the translation aims to achieve 0% information loss during the translation so all MESP data types are mapped to an NGSI data object. A first obvious outcome from this process is the increase of the overall data volume. According to the FIWARE-GSMA vocabularies the CO_2_, Air Humidity, Air and Soil Temperature information elements can’t be modelled under the same NGSI Entity. In order to be consistent with the already defined FIWARE models the respective MESP sensed data need to be modelled as Attributes of the Entities of type “AirQualityObserved” and “GreenSpaceRecord”. In addition, the raw measurement from the flame detection sensor can’t be modelled with the existing NGSI Attributes hence the Attributes of the Alert Entity are utilised. Toward this scope an inference process is necessary in order the raw flame sensor measurement to be mapped to a higher concept modelled as Alert [63] which is associated with the following classes:, informational, low, medium, high, critical. More details on the inference algorithms are presented in Section 5.3.

### 4.3. Integration of Social Media Data with NGSI

As it was analysed in Section 2.2, Social Media (SM) utilisation by large population proportions dictates a new data gathering paradigm also known as participatory social sensing. However, well established initiatives such as FIWARE are not yet officially foresee the utilisation of such information sources even though such objectives are already pursued from the research community [64]. Within the scope of this article it is considered that the time is ripe for the integration of social networking concepts with NGSIv2 API and data model hence a proposal to map core social media information aspects to NGSI is introduced. However, it should be noted that this integration effort doesn’t aim to substitute the social media information systems by IoT platforms so only core social information elements are currently targeted. In addition, and aiming to achieve a minimum viable interoperability level this integration approach can be considered as a first step that will be feasible to build upon and further extend it. To this end, two new NGSIv2 Entity types are introduced namely the: “SocialMediaContent” and “SocialMediaUser”. The “SocialMediaContent” is assigned with the mandatory NGSIv2 attributes (type and id) and also with a set of social media specific attributes that are commonly present to the dominant platforms such as Facebook and Twitter. These attributes aim to be generic enough and able to represent publicly shared user content. Analysing the data structures of the Twitter and Facebook platforms we identified common core modelling approaches.

As is presented in Table 2, these platforms adopt similar semantics for posted messages, the number of times a message was “liked” and/or “shared” and the “tags” that a message may contains. Building on this common semantics we introduced the respective NGSIv2 Attribute types. In addition, the SocialMediaContent contains attribute types about the time a message was created, the URL of the originating platform, the language utilised, etc.

**Figure 9:**
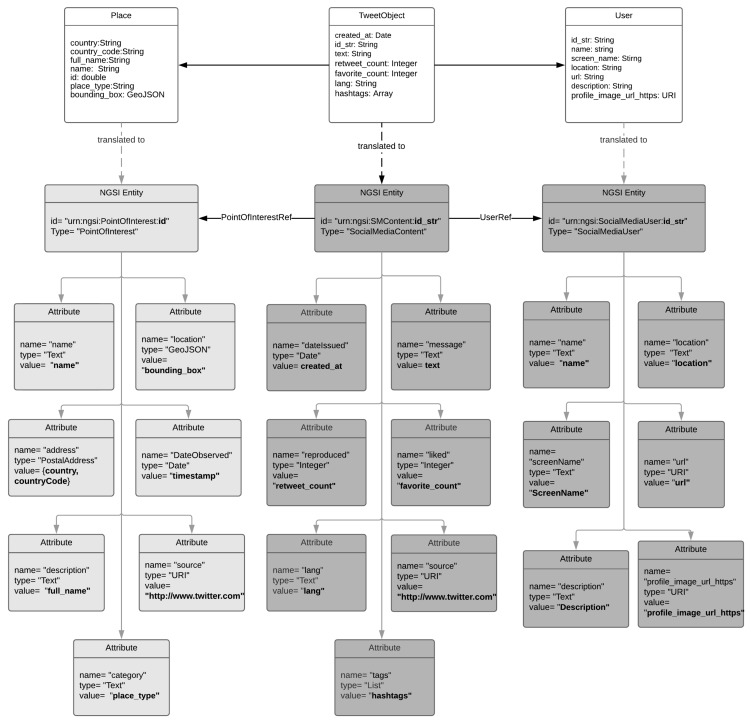
Mapping Twitter data objects to an NGSIv2-based information model.

The second introduced entity, “SocialMediaUser”, aims to represent profile information of the social media user. Again, the introduced attributes are the minimum common outcome of an analysis performed on the dominant social media platforms and the information that are maintain about user’s profile. In Figure 9, an illustration of this mapping is presented. Introduced data object are coloured with dark grey while reused NGSI data objects with light grey. 

The actual retrieval of the social media information elements is realised through the utilisation of the respective social media APIs. It should be noted that there are various limitations imposed that need to be carefully addressed. For example, the standard version of Twitter API enforces “Rate Limits” allowing a certain number of calls on 15 min intervals. In a similar manner, Facebook enforces strict privacy protection policies which are limiting the amount of information that can be retrieved while these policies are subject to frequent updates which services consuming the APIs should follow.

## 5. Experiment Design and Rationale

### 5.1. Experiment Rationale

In order to evaluate the feasibility of the proposed cross-domain data interoperability mechanisms the application domain of emergency detection and evaluation is selected as a demanding use case where information richness is crucial for the early assessment of the overall severity of the situation. A combination of climate change, population growth, negligent forest management and modern building practices has had an increasing effect on the proliferation of forest wildfires at a global level over the last few years [65]. From the “national tragedy” that hit Greece in the summer of 2018, with the worst wildfire in over a decade killing 100 people, injuring over 200 and leaving thousands more homeless in Rafina-Mati area, a small resort town near Athens, to the “deadliest and most destructive fire in the USA’s modern history” in California, where more than 150,000 acres of land were burned and 86 casualties were recorded, in November of the same year, it is made clear that the fire season now lasts longer, until the end of fall and the wildfires tend to share some common traits; flames are fueled by dryness and drawn by strong winds towards populated areas with clogged escaped routes and little advanced warnings. 

Regarding the latter, leveraging the recent technological progress made in the sensing and networking areas, massive effort has been put into monitoring and detecting abnormalities [66,67] in order to assist the early alert of the civilians in proximity and the rapid suppression, before they become uncontrollable. Taking also advantage of the fact that these fires usually break out geographically close to semi-rural areas, extensive coverage from cameras (e.g., smartphones, home security and outdoor surveillance cameras) and data from social media become instantly available, thus, together with the latest innovations in the 5G networks, Internet of Things and Big Data analytics, the firefighting technology is enabled to adopt solutions from the smart computing and cyber-physical systems domains so as to acquire, process and combine this available information and act accordingly.

### 5.2. Experimental Setting

**Figure 10:**
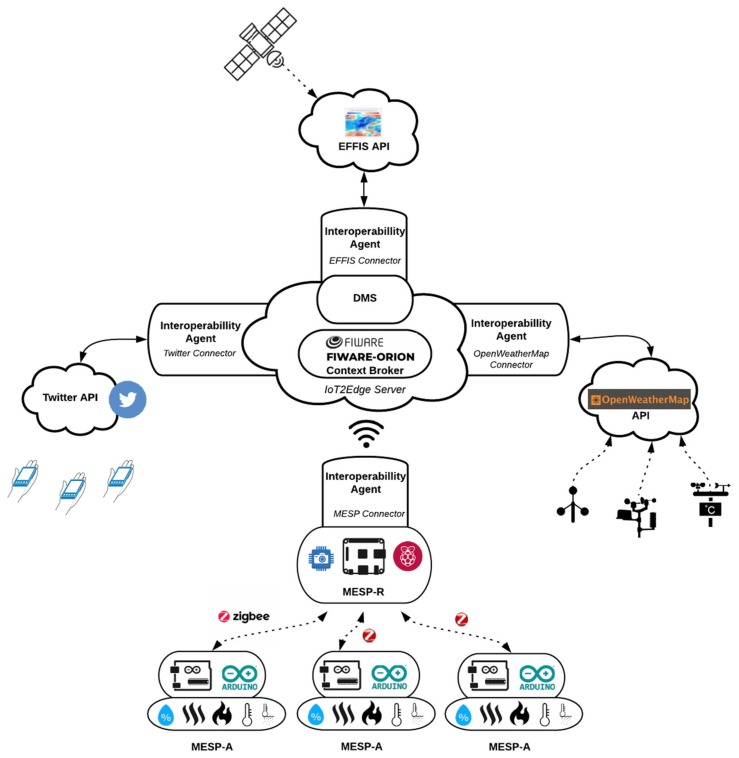
Deployment of IoT2Edge data interoperability modules for retrieving data from information platforms participating to the experiment.

This experiment aims to evaluate the resulting performance of the emergency incident detection and monitoring mechanisms enabled by the adoption of the IoT2Edge data interoperability approach. For the evaluation of the proposed mechanisms, heterogeneous platforms that contribute to the information completeness enhancing the performance of the dynamic decision making engine are engaged. The decision engine is expected to automatically assess how critical the emergency situation is, based on a predefined scale (i.e., low, medium, high). It is foreseen that the confidence and promptness of the reasoning outcome will be higher and more accurate as the richness of the available information increases. In this approach, the following benefits are expected to be demonstrated: data interoperability of the IoT and social media services in support of cross application domain reasoning, through the homogenisation of information and automated identification of an emergency situation. The experiment envisages a semi-rural area where various information platforms are able to collect a wide variety of data related with local environmental parameters, social media activity, weather forecast, and fire danger index. In more detail, and as illustrated in Figure 10, the following platforms are considered:

The intelligent Mobile Environmental Sensing Platform (MESP): The MESP aims to advance IoT platforms by introducing intelligent behavior and actuation in the process of data sensing. It consists of a unique central gateway realised on Raspberry Pi 3 B+ platform, called MESP-R, that acts as an aggregator of data collected from various Arduino platforms called MESP-A. 

**Figure 11:**
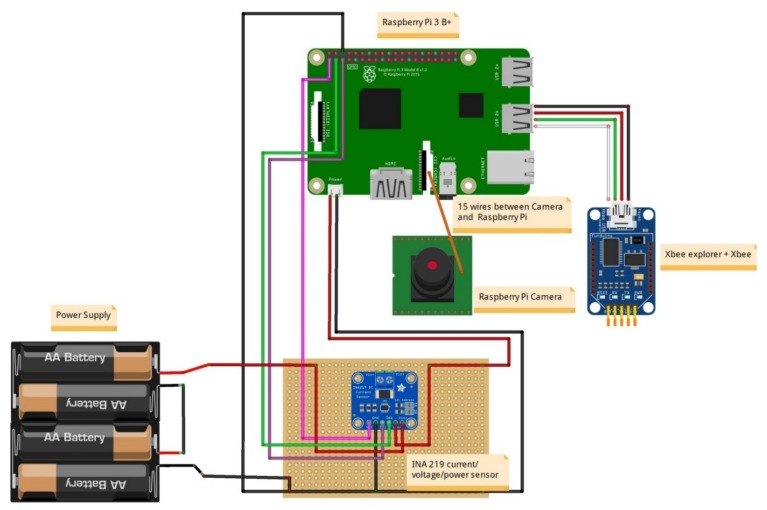
The MESP-R consists of a Raspberry Pi board with attached modules supporting communications, optical sensing and power consumption monitoring.

MESP-R: A Raspberry Pi 3 Model B (https://www.arduino.cc/en/Main/Products) operates with the latest operating system “2018-11-13-raspbian-stretch-full”, while communication with the MESP-A is facilitated through a Xbee Zigbee S2 PRO 63 mW with an embedded wire antenna module. The Xbee module can achieve a range of 2 miles on a Line-of-sight, with a maximum bitrate of 250 kbps, which is more than sufficient for our experiment setting. In addition, a Raspberry Pi Camera IR-CUT is attached to the MESP-R, which is equipped with a 5 Megapixel OV5647 sensor. The sensor’s maximum resolution is 1080p and supports the connection of an IR LED, so that it can operate in a low lux (night vision) environment. Finally, an INA219 current sensor is attached for measuring voltage, current and power consumption utilised for the evaluation of the introduced mechanisms. Figure 11 illustrates the setup of the MESP-R in detail.

**Figure 12:**
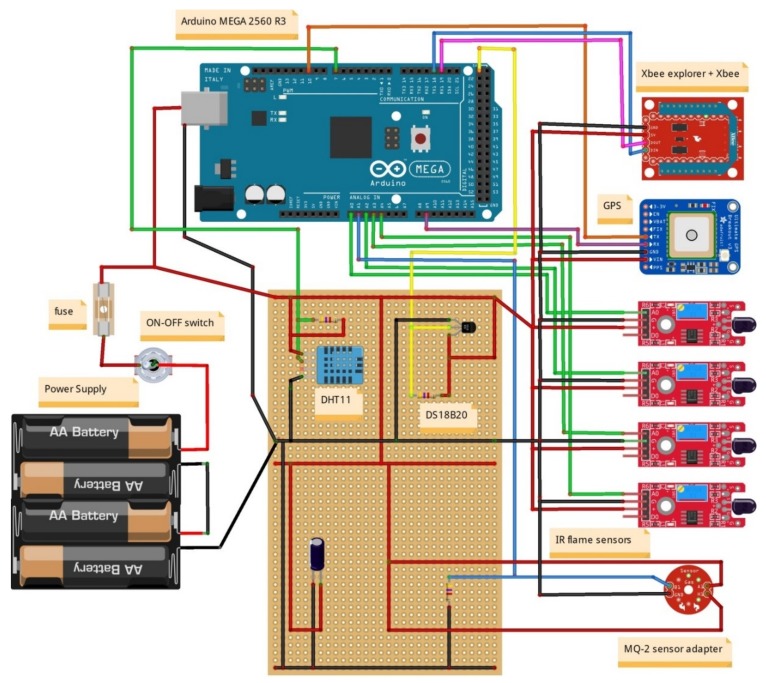
The MESP-A consists of an Arduino board with attached communication and environmental sensing modules.

MESP-A: The core component of the MESP-A is an Arduino MEGA 2560 R3 (https://www.arduino.cc/en/Main/Products) which performs the aggregation, processing and communication of the sensed data. Attached there are 4 IR flame sensors, which operate on the IR spectrum range between 760 nm and 1100 nm, with a 60-degre detection angle. An MQ-2 Gas Sensor Module, which is sensitive to LPG, propane, hydrogen, and smoke, is also attached. Its output voltage boosts along with the concentration of the measured gases increases. MESP-A also consists of a DHT11 Sensor, which is used for detecting ambient temperature and humidity through a standard single-wire interface. For measuring soil temperature, we use the DS18B20 waterproof temperature sensor. Location information is recorded through the attached Adafruit Ultimate GPS Breakout version 3. Communication with the MESP-R is realised through an Xbee Zigbee S2 PRO 63 mW with an embedded wire antenna. Finally, a rocker ON-OFF switch is attached to the project box which enclosed the MESP-A. Energy is supplied to the system by a power bank 5.1Volt/2.5 Amperes with 6.900 mAh. Figure 12 illustrates the setup of the MESP-A in more detail.

The rationale for constructing the MESP device is to have a multi-purpose micro-environmental sensing prototype useful for testing and evaluating various deployments and data management approaches. MESP demonstrates both the capabilities as well as the limitations of IoT platforms, hence it is considered as an appropriate test system for assessing different IoT-related mechanisms, e.g., the described data interoperability mechanisms. Within the scope of the wild-fire detection scenario, MESP operates as a sensing device deployed in the perimeter of a house located at a semirural area, i.e., a house surrounded by trees, close to a forest (as was the case in Rafina’s forest-fire and California’s camp fire incident). The camera controlled by MESP simulates a CCTV system monitoring the perimeter of the house. For the needs of the described experiment an IoT2Edge-Agent was deployed on MESP-R in order to retrieve, translate, process and disseminate the recorded environmental data.

OpenWeather Map API: The OpenWeatherMap (https://openweathermap.org/) is an open platform that provides current and forecasted weather information through API calls. Within the scope of the conducted experiment, this API was utilised for accessing current weather data for locations specified through their GPS coordinates. OpenWeatherMap platform aggregates weather data from more than 40.000 stations deployed all over the planet while the results are provided in many forms (e.g., JSON, XML, HTML). For the needs of the described experiment, an IoT2Edge-Agent was connected to the OpenWeatherMap API in order to retrieve current and forecasted weather data for a specific location.

EFFIS API: The European Forest Fire Information System (EFFIS) [68] offers updated and reliable information services about wildland fires in Europe. The EFFIS service provides maps for active fires and burnt areas for a specific number of past days, while it also provides fire danger forecasts. These forecasts are based on the Canadian Forest Fire Weather Index (FWI) System where past, current and future weather information are aggregated with location specific information (e.g., history of past fires, availability of fuels in the area, vegetation, etc.) and they are generated as daily maps using numerical fire danger predictions encoded in GeoTIFF maps. Fire danger is mapped in 6 classes (very low, low, medium, high, very high and extreme) with a spatial resolution of about 16 km. The EFFIS FWI data are retrieved by a dedicated IoT2Edge-Agent module through the respective API.

Twitter API: For the needs of this experiment the Twitter micro-blogging platform has been integrated. Twitter maintains a total number of 335 million monthly active users, who produce more than 500 million Tweets per day. The fact that 80 percent of Twitter users utilise the service through mobile devices, makes this social network an ideal platform for promoting the social sensing paradigm. In addition, Twitter provides an open, well specified API with almost unrestricted access to the publicly available, user-provided content and profile information. Data collection for the execution of this experiment is based on criteria expressed as hashtags and keywords associated with wildfires, combined with tags denoting geo-reference. Twitter also offers the option to geo-tag the provided Tweets, but this feature is not frequently used as it is a common practice for Twitter users to introduce their own tags in order to express the connection of their posts with a specific area. To this end, text analytics approaches have been employed as more appropriate. Again, an IoT2Edge-Agent has been deployed for retrieving and translating relevant with the experiment Tweets. 

### 5.3. The Decision-Making Process and the Knowledge Extraction Services

Beyond the representational aspects, semantic computing supports reasoning on raw sensor data enabling the derivation of higher level abstractions; such abstractions form the basis of domain- and cross-domain knowledge which is the main enabler for decision making. To this end, and as it is described in Section 4.1, the IoT2Edge-Agent “Inference Service” provides the necessary mechanisms in order to assign the appropriate domain concepts to the respective data collections. These rules are expected to be tailored to the needs of each application domain and the specifics of the collected data. For the needs of the wild-fire detection use case and for the realisation of the respective experiment, the following inference processes have been identified for each information platform:

MESP data classification: Sensors measurements and images collected by the MESP are combined in order to deduce higher level alert information for the potential identification of a fire ignition in the area. A custom image classification service is deployed as a native standalone application on MESP-R, in order to classify images as “fire-containing” or not. This service, which was initially developed for the purposes of the work presented in [69], leverages Google’s Tensorflow deep learning framework. The “Inception v3” Deep Neural Network was retrained with a specific dataset [70] containing either pictures of forest wildfires or plain forests. Images captured through the MESP-R camera are assigned a score ranging from [0,1], where 1 corresponds to the absolute confidence about the presence of a fire in the image. The numerical image classification scores are combined with the measurements derived from the infrared, temperature, gas monitoring and humidity sensors through data inference algorithms such as those presented in [45,47]. The aggregated outcome is normalised within the [0,1] range of values. Table 3 contains the measurements range for each MESP sensor along with indicative thresholds denoting the presence of a fire.

The outcome of the inference process is modeled based on the 5 FIWARE Alert information classes (Informational, Low, Medium, High, Critical) [63]. An example of the Alert JSON object produced by the MESP data classification engine is presented hereafter:

{
 "id": "Alert:1",
 "type": "Alert",
 "category": "security",
 "subCategory": "forestFire",
 "severity": "high",
 "location": {
  "type": "Point",
  "coordinates": [23.7319, 37.9877]
 },
 "dateIssued": "2018-07-23T09:25:55.00Z",
 "description": "Potential forest fire identified by MESP",
 "alertSource": "https://iot2edge.ece.ntua.gr/mesp/"
  }

Tweets Classification: The developed inference engine processes the messages contained in collection of Tweets aiming to identify time based changes on the utilisation of specific terms. In our case, the Twitter API is utilised in order to retrieve Tweets containing tags and keywords referring to the targeted locations along with fire-related keywords potentially denoting the ignition of a forest fire incident. Collected tweets are periodically processed at regular time intervals and an Alert class is assigned to them, based on textual content analysis. In a nutshell, the rationale of the algorithm that extracts the probability of a fire incident and the respective fire alert level based on information originating from Tweeter, is as follows: initially, the population $T_{L}\left(wf_{i}\right)$ of the retrieved tweets posted in the latest window frame ($wf_{i}$) that use keywords linked to the area of interest (this is expressed by the keyword set *L* that results from a reverse geocoding process for the provided GPS coordinates, which uses external APIs such as the Google Maps API), is measured. For example, for the studied fire incident, the respective keyword set was as follows: *L* = {*rafina*, *mati*, *penteli*, *dionisos*} Subsequently, the population $T_{LF}\left(wf_{i}\right)$ of the subset of $T_{L}\left(wf_{i}\right)$ is measured. This counts the tweets posted in the latest window frame ($wf_{i}$) that use both keywords linked to the area of interest, as well as keywords related to fire emergencies (this is expressed by the keyword set *F* that is language dependent, aggregating fire emergency keywords expressed in the official language(s) of the country where the area of interest is located at). For example, for the studied fire incident, the respective keyword set was as follows: *F* = {*pyrkagia*, *fotia*, *emprismos*, *kapnos*, *kaigomaste*}. Finally, the ratio of the two aforementioned metrics is calculated: $T_{score}^{L}\left(wf_{i}\right)=\frac{T_{LF}\left(wf_{i}\right)}{T_{L}\left(wf_{i}\right)}$, the value of which lies in the range [0,1]. The respective translation of $T_{score}^{L}\left(wf_{i}\right)$ to a Twitter-based fire alert level $T_{FAL}^{L}\left(wf_{i}\right)$ is executed as follows:

$$T_{FAL}^{L}\left(wf_{i}\right)=\{\begin{array}{cc} \mathrm{informational} & ,\;\mathrm{if}\;T_{score}^{L}\left(wf_{i}\right)\in \left[0,0.2\right)\\ \mathrm{low} & ,\;\mathrm{if}\;T_{score}^{L}\left(wf_{i}\right)\in \left[0.2,0.4\right)\\ \mathrm{medium} & ,\;\mathrm{if}\;T_{score}^{L}\left(wf_{i}\right)\in \left[0.4,0.6\right)\\ \mathrm{high} & ,\;\mathrm{if}\;T_{score}^{L}\left(wf_{i}\right)\in \left[0.6,0.8\right)\\ \mathrm{critical} & ,\;\mathrm{if}\;T_{score}^{L}\left(wf_{i}\right)\in \left[0.8,1\right] \end{array}.$$

More details for the respective mechanism are presented in [71]. An alert level based on the FIWARE Alert information model [63] is associated with a location and a time interval. An example of the Alert JSON object produced by the Twitter data classification engine is presented hereafter:

{
 "id": "Alert:2",
 "type": "Alert",
 "category": "security",
 "subCategory": "forestFire",
 "severity": "high",
 "location": {
  "type": " Polygon",
   "coordinates": [23.7319, 37.9877]
 },
 "dateIssued": "2018-12-12T09:25:55.00Z",
 "description": "Potential forest fire identified by Tweet analysis",
 "alertSource": "http:/www.twitter.com"
}

Weather Classification: Weather related measurements collected from data sources deployed in the area are processed and the respective fire weather index (FWI) is extracted, based on the Canadian forest fire danger rating system [72], which is one of the most popular models used to link weather conditions with the probability and risk of wildfire incidents. The estimated FWI is normalised according to the FIWARE Alert information model severity classes [63]. An example follows:

{
 "id": "Alert:3",
 "type": "Alert",
 "category": "weather",
 "subCategory": "fireRisk",
 "severity": "high",
 "location": {
  "type": " Polygon",
   "coordinates": [23.7319, 37.9877]
 },
 "dateIssued": "2018-12-12T09:25:55.00Z",
 "description": "Increased fire risk indicated by weather data analysis",
 "alertSource": "http://www. openweathermap.com"
}

EFFIS classification: The EFFIS alerts are already classified based on a scale of six levels (http://effis.jrc.ec.europa.eu/about-effis/technical-background/fire-danger-forecast/) that are directly mapped to the FIWARE Alert information model severity classes [63].

**Figure 13:**
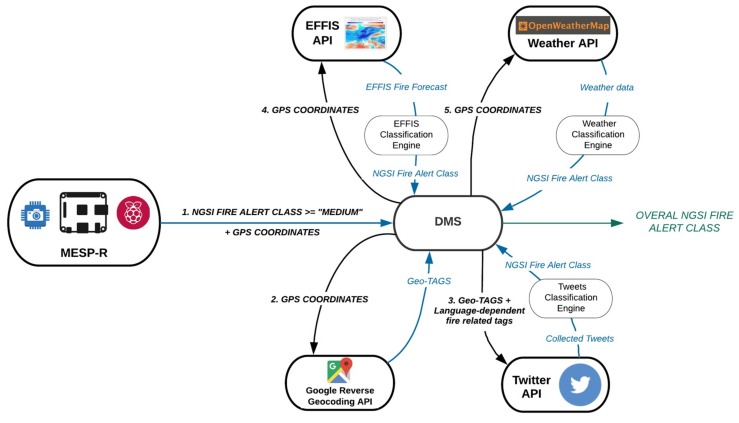
The sequence followed for the decision-making process.

As illustrated in Figure 13, the Decision-Making Service (DMS) is deployed to the cloud as an IoT2Edge-Server component and is triggered by the MESP system when the fire-related average classification score of the images and the environmental sensors is above a predefined threshold, e.g., “Medium”, thus indicating a considerable probability of fire occurrence. The overall operation can be triggered periodically and runs continuously, meaning that the DMS may receive multiple fire alerts from various locations, where a decision should be extracted for each case. Once the DMS is triggered, it aggregates the various aforementioned data, in order to further evaluate the probability of a fire incident occurrence, the severity of the potential fire incident (e.g., in terms of human presence in the area, potential fire spreading, etc.). As it is already described, the alert levels derived from the diverse environmental monitoring and social media management platforms are modelled in uniform alert classes and are propagated to the cloud-based decision making service that is responsible for processing the respective values and generating an overall indication of the fire risk level.

Among the benefits of this approach is that no sensitive information (e.g., images) is disseminated from the respective distinct cyber-premises and only the alert levels are provided to the DMS, realising a privacy by design approach. In addition, the utilisation of common semantics through the enforcement of the data interoperability mechanisms facilitates the rapid cross-domain decision making.

In a nutshell, the DMS delivers the respective outputs based on the following steps that take place:

Step 1: The MESP system sends a fire alert notification (if the respective level is “MEDIUM” or higher), along with the associated location expressed in GPS coordinates to the DMS.

Step 2: The DMS executes a reverse geocoding process for the provided GPS coordinates, which uses external APIs (e.g., Google Maps API) in order to obtain a set of area names and location identifying keywords.

Step 3: The DMS triggers the Twitter-based Fire Detection Service with these keywords that collects and analyses the Twitter posts that are related to the indicated area, then identifies the ones that are potentially associated with an emerging wildfire incident and finally extracts the probability of a fire incident occurrence that is eventually returned to the DMS.

Step 4: The DMS then triggers the EFFIS-based Classification Service with the specific GPS coordinates, which retrieves GeoTIFF image provided by the EFFIS and extracts the Fire Danger Class for the provided coordinates that is then returned to the DMS.

Step 5: The DMS uses the Weather-based Fire Danger Classification Service that collects and analyses current and forecasted weather data that are linked with the indicated area (via the OpenWeather Map API) and uses them to extract the fire risk scores that are then returned to the DMS.

Step 6: The DMS processes and fuses the outputs of the four classifier services above in order to extract estimations for two fire-related metrics for the specified GPS coordinates: (i) the overall fire danger level (indicating the risk of fire ignition) and (ii) the probability of fire occurrence. More details on the internals of this step are provided in [73].

## 6. Experiment Execution and Evaluation of the Findings

Two families of experiments have been conducted to evaluate the proposed solution. The first set is elaborated upon in Section 6.1 and aims to validate the stand-alone data interoperability mechanisms especially focusing on the MESP and social media interoperability mechanisms. The second one is discussed in Section 6.2 and aims to validate the proposed cross-platform decision support mechanism that exploits IoT, weather and Social Media data in order to extract knowledge regarding the risk of wildfire occurrence or wildfire incident probability.

### 6.1. Validation of MESP and Social Media Data Interoperability Mechanisms

The first experiment was realised by deploying the IoT2Edge-Agent interoperability enabler on the MESP-R platform. Data collected from MESP-A where sent to MESP-R and then retrieved by the “Connector” component of the IoT2Edge-Agent module. The sensor reading was then translated to NGSIv2 and sent through a REST call to the Orion Context Broker (OCB) deployed on IoT2Edge-Server. For evaluating the introduced overhead, a first set of 1000 measurements was conducted by using the native MESP data objects that were sent to the OCB. A one minute interval was introduced between each measurement. Subsequently, 1000 MESP measurements were translated to NGSIv2 according to the mapping illustrated in Figure 7 and in a similar way for NGSI-LD according to the mapping in Figure 8.

**Figure 14:**
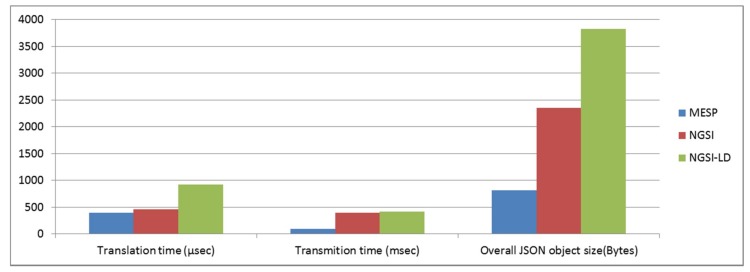
Average data translation time, average data transmission time and overall JSON object size for the original MESP, NGSI and NGSI-LD models.

As it is depicted in Figure 14, the interoperability enabler introduces an overhead that affects both the transmission time and the overall transmitted data volumes compared with the non-interoperable natively modeled MESP measurements. It should be noted that there occurred no information loss which means that the total of the MESP measurements were translated and transmitted to the cloud services. This is due to the fact that the translation process is designed in a way that maintains the full information set of the original recorded measurements. All the initiated calls where successfully performed, while an HTTP Success Status Code confirmation was received for each REST call by the IoT2Edge-Agent. In addition, the average energy consumption of the Raspberry Pi was measured at 3850 mW, while, when the IoT2Edge-Agent was deployed and operating, there was an increase to 5174 mW. Given that the utilised power source of the MESP-R is a power bank with the following characteristics: {5.1 Volt, 2.5 A, 6.900 mAh}, in the first case the battery lasted for 8 h 57 min and 39.6 s, while in the second case 6 h 40 min and 1.2 s. This means that the employed data interoperability mechanism causes the MESP-R to operate approximately 2 hours and 18 min less.

**Figure 15:**
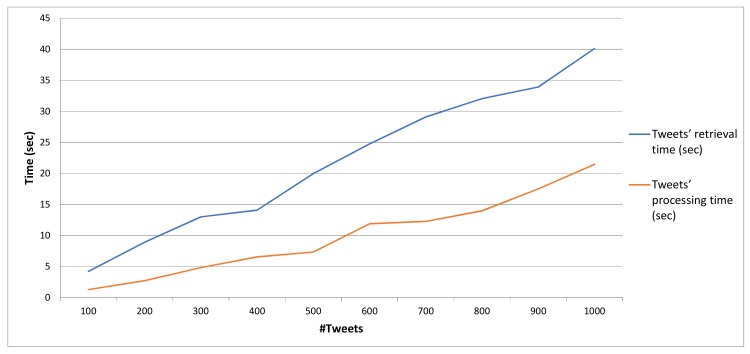
Time required to retrieve related Tweets using the Twitter API and overall Tweet processing time (i.e., time to clean, translate and post NGSI-modeled Tweets to a remote instance of OCB, where interoperability overhead is introduced) against the individual Tweet populations.

With regards to the social media integration, the evaluation was performed with the IoT2Edge-Agent’s Social Media connector targeting the Twitter service. The evaluation included the performance benchmarking of the Tweet data objects retrieval, preparation of data, translation to the introduced NGSIv2 data model and posting to Orion Context Broker (OCB). The preparation phase included, among others, the cleansing of tweets from characters that are not supported by the OCB such as the < > “, = ; and ( characters. Figure 15 below summarises the time needed to retrieve, process and store increasing data sets of Tweets.

The results show that the adaptation of NGSIv2 and OCB for managing and storing data derived from social media services is feasible with a minimum overhead compared with the amount of information processed. For the conducted experiments the average size of a native tweet object was calculated to 25,017.6 characters, which corresponds to different byte sizes depending of the encoding. The translated NGSI Tweet object contained 921.3 characters on average. As noted, there is a significant information loss. However, this is dictated by the fact that native Tweet objects embeds numerous information elements and metadata regarding the user profile’s preferences (e.g., profile image, selected colors, user bio, device used for tweeting, etc.). This kind of information is considered as redundant for the application domain targeted in this article. Nevertheless, these information elements are still retrievable through the Twitter API and are based on the Twitter user ids that are stored within the NGSI-Tweet. An example of a JSON formulated according to NGSIv2 specification is available in Appendix A.

A critical limitation that the OCB demonstrates is that it is not able to handle data objects larger than 1 MB. This is due to the fact that the OCB is designed to operate as an IoT data platform, hence it is capable of managing simultaneously occurring updates that demonstrate high rates but small data footprint. This design characteristic does not allow the storing of multimedia files which have a significant role in social media, e.g., pictures posted by the users. However, various workarounds exist, such as utilising and storing the URLs that to the multimedia files.

### 6.2. Evaluation of Cross-Domain Decision Making

The second set of experiments aims to validate the proposed cross-platform decision support mechanism through the exploitation of diverse information sources. As it was mentioned in Section 5.1, at 16.40 on 23 July 2018, a lethal wild fire broke out at the eastern part of Attica Greece, 20 km from Athens city center in a semi-rural touristic area called Rafina-Mati. The outcome of this fire was disastrous with a large number of fatalities [74]. As this major incident drew the attention of media, it was feasible to retrospectively collect valuable data and utilise them for evaluating the interoperability and decision making approaches introduced in this article. To this end, social media posts, video streams, and weather data were collected and different IoT2Edge-Agents where employed in order to process and translate this data according to the NGSIv2 data model.

With regards to the social media, the first related tweet was posted at 16:53, is publicly available and it states (translated from Greek) “We are burning…..!!!!! Rafina Ntaou Pentelis right now, God help us today mercyyyy!” [75]. This tweet contains tags (“Rafina” and “Ntaou Pentelis”) that specify the exact location of the fire and also keywords that allow the deduction of the severity of the situation. It is also worth noting that the tweet was posted just 12 min after the official ignition of the fire. Using the Twitter API, we collected all the Tweets from 19/7/2018 until 26/7/2018 that contained Greek keywords linked to the targeted area (e.g., “rafina”, “mati”) and keywords related to fire emergency situations (e.g., “fire”, ”conflagration”, ”blaze”). The respective data are summarised in Table 4.

**Figure 16:**
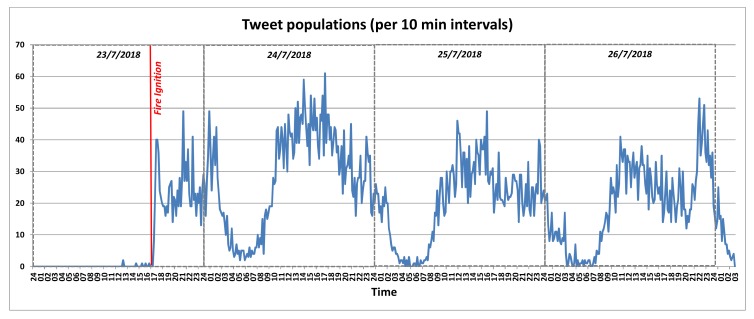
Tweet populations that are linked to the Mati-Rafina fire incident on 23/7/2018, aggregated over 10 min intervals.

More fine-grained analysis of the related Tweets collected on a basis of 10-min intervals, is illustrated in Figure 16.

As one may easily observe, the moment the fire started, the tweet populations started to increase promptly. The periodic nature of the tweet populations’ fluctuation is also apparent, as during the night the tweet populations drop drastically, reaching their peak at noon or afternoon during the days that follow the fire ignition. As analysed in Section 5.3, the streams of tweets were processed, Alert messages were generated and stored in the Orion Context Broker and fed to the Decision Management Services.

**Figure 17:**
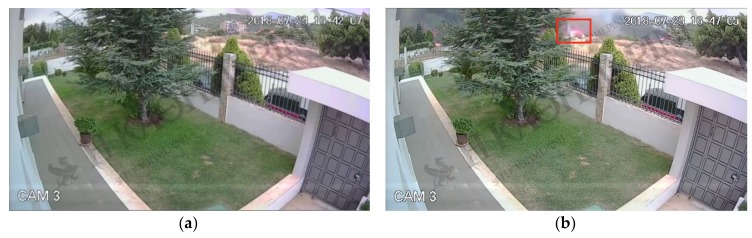
Two sample snapshots capturing the start of the Mati-Rafina fire incident on 23/7/2018: (**a**) when the fire is still far from the specific premise and (**b**) 5 min later, when the fire is closer and the flames are well visible.

A CCTV camera monitoring a house yard in the area accidentally captured the ignition of the fire. The video was made publicly available by the press [76] and is also posted in YouTube (https://www.youtube.com/watch?v=MokHB__1ynY). As it is shown in the video, the first fire smoke is visible at 16:41 and, according to the media, fire brigade services where notified at 16:49. The video was decomposed to 173 images that were fed to the MESP classification engine. The video covers a time frame from 16:40 until 16:57. However, it can be safely assumed that prior to the fire ignition, the captured images generate a score similar to that of the first minutes of the video recording. Sample of the respective snapshots captured are presented in Figure 17.

**Figure 18:**
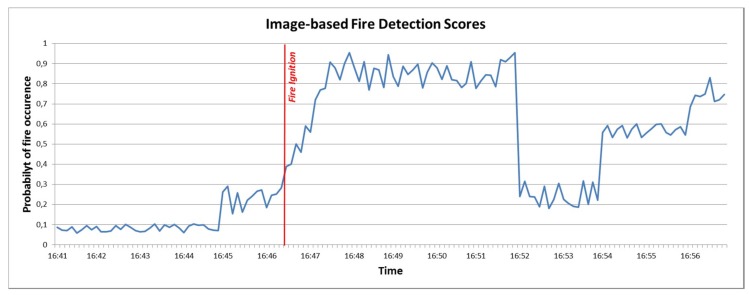
Image-based fire detection scores for the Mati-Rafina fire incident on 23/7/2018 over a 17 min interval before and after the actual fire ignition.

The extracted alert scores are presented in Figure 18 below. It is evident that the scores rapidly increase upon fire ignition and remain very high for several minutes. The fact that they drop drastically at some point is attributed to the strong winds that completely changed the course of fire, thus saving the actual house the camera of which captured the processed video. 

Finally, the fact that the fire detection score is increased again after a few min, is due to the fact that heavy smoke was captured by the camera, though it was observed in a larger distance. Based on the “MESP data classification” engine, the image based fire scores were classified based on the FIWARE Alert information model severity classes.

Weather condition measurements regarding air temperature, relative humidity, wind speed and precipitation have been collected from local weather stations the days before, during and after the specific fire incident. A sample of the weather related measurements for the time of the day that the fire occurred in the specific area, are presented in Table 5.

**Figure 19:**
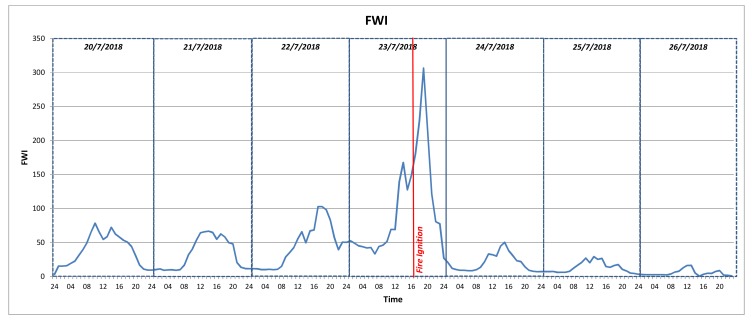
Fire Weather Index for the Mati-Rafina fire incident in Greece (23/7/2018) extracted based on environmental condition measurements obtained by local weather stations.

As mentioned earlier, the weather measurements have been processed to extract the respective fire weather index (FWI), based on the Canadian forest fire danger rating system. The diagram in Figure 19 depicts the respective FWI extracted. 

As one may easily observe, the fire ignition occurred when very high fire weather index levels were in place (i.e., above 100) and for the entire fire duration the respective FWI remained extremely high (it is noted here that based on the EFFIS Fire Danger Class mapping to FWI ranges (http://effis.jrc.ec.europa.eu/about-effis/technical-background/fire-danger-forecast/), the Extreme Fire Danger Class (most dangerous in the EFFIS scale) is mapped to FWI above 50). Based on the IoT2Edge-Agent “Weather Classification” engine these measurements were normalised according to the FIWARE Alert information model severity classes.

**Figure 20:**
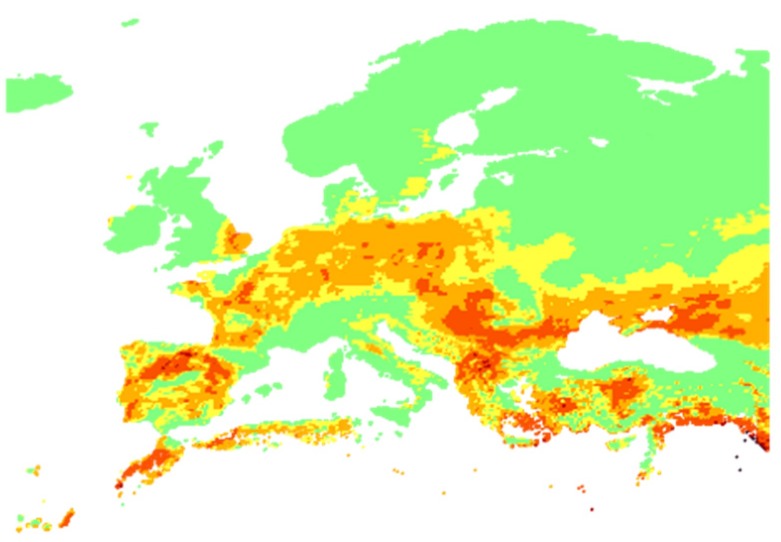
The fire alert levels embedded with different colors on a GeoTIFF raster image provided by EFFIS service for 23/7/2018 across Europe.

Finally, the alert level provided from the EFFIS system for 23/7/2018 was classified as “HIGH” for the specific area studied in this evaluation, which is also visible in Figure 20.

As already elaborated upon in Section 5.3, the DMS, once triggered by the MESP, uses the various fire classifier services (MESP, weather-based, social media-based, EFFIS-based) to extract the two metrics of interest. Regarding the fire danger level, the DMS classifies the conditions at the highest possible level the entire day when the fire incident occurred. The input used for this purpose relies on local weather stations and EFFIS data. Regarding the fire occurrence probability, experiments have been conducted using the processed data obtained for the Mati-Rafina fire incident as input. A simple linear weighted formula has been employed by the DMS to detect potential fire incidents, using as input the following (in priority order): MESP (image), Social Media, Weather, EFFIS. Thus, the fire detection formula of the DMS can be expressed as follows:

$$FD_{score}^{L}\left(wf_{i}\right)=c_{MESP}\cdot MESP_{score}^{L}\left(wf_{i}\right)+c_{T}\cdot T_{score}^{L}\left(wf_{i}\right)+c_{W}\cdot W_{score}^{L}\left(wf_{i}\right)+c_{EFFIS}\cdot EFFIS_{score}^{L}\left(wf_{i}\right)$$

where the four scores lie within the range [0,1] and express the fire alert levels extracted from the four inputs afore mentioned. The same stands for the values of the respective weights, for which it also stands: $c_{MESP}+c_{T}+c_{W}+c_{EFFIS}=1$ and $0<c_{MESP}<c_{T}<c_{W}<c_{EFFIS}<1$ to reflect the priorities previously mentioned. The respective detection of potential fire incident has been estimated as follows: in case $FD_{score}^{L}\left(wf_{i}\right)$ lied above a redefined threashold *FD_TRUE_*, then the DMS raised a fire detection alert, else it does not fire any alert.

To validate this approach, the DMS was triggered to detect potential fire incidents every two min for the period 20–26/7/2018. The DMS outcome should be positive for the period 23/7/2018 (16:41)–23/7/2018 (22:30) and negative for the rest of the studied duration. This resulted in over 5000 DMS triggers for 93.6–98.9% of which the DMS successfully detects the existence or lack of fire incident occurrence for the optimal weight configurations, where the range of *FD_TRUE_* values tested was [0.60,0.95]. It should also be noted, that the DMS succeeds in detecting the fire incident only a few minutes after the actual fire ignition. The input used for the extraction of this knowledge relies on MESP and Social Media data. An indicative subset of the various IoT2Edge classifier services outputs regarding the fire danger level and the probability of fire incident occurrence is presented in Table 6 below.

The benefits gained by having available all critical information at one place are evident; a command and control center is able to take the right decision at the right time, while it is feasible to be notified with valuable details (e.g., exact location, human presence, potential for future evolution of the incident) of a critical situation, in an automated manner and without the need to utilise human resources for manually monitoring all these information sources. Based on the results of this experiment, it is also evident that data interoperability enablers are able to realise the aggregation of IoT and social media services in order to facilitate cross-domain decision making.

## 7. Conclusions and Future Work 

This article elaborates on data interoperability in IoT systems and proposes a solution towards the support of uniform data exchange to enable cross-platform and cross-domain decision making. As indicated by the findings presented in this article, the realisation of IoT data interoperability is well feasible, while the respective potential benefits are huge and go way beyond the economic added value generated from innovative service to life saving operations. Among the core design principles of the IoT2Edge-Agent interoperability enabler is to use widely accepted de-facto standard semantic and syntactic approaches aiming to realise a minimum level interoperability approach. In addition, the IoT2Edge-Agent supports the semantic annotating of sensed data through inference techniques which facilitates the cross-domain decision support but also avoids the transmission of larger data volumes such as video recordings and raw data measurements. This feature also contributes to the protection of the privacy of individuals by keeping sensitive data (e.g., images and video recordings) within the cyber-premises of the data owning entity and exposing only the necessary aggregates. 

This article also introduces the required semantics and the respective data processing mechanisms for integrating social media data within the NGSIv2 data model and API, while to the best of the authors’ knowledge this is the first time that such a utilisation approach is specified. The importance of this integration is that IoT and social media are modeled following the same widely accepted (de-facto standard) information model and API thus facilitating the realisation of advanced interoperable services.

Experiments conducted and presented in this article led to significant findings regarding the performance of the proposed framework. In addition, during the experiments the authors had the opportunity to get familiarised and evaluate the introduced improvements by the NGSI-LD ETSI standard that will be officially announced on March 2019. The experiments also demonstrate the benefits of aggregating information from diverse sources towards advanced decision making in the scope of the early detection and monitoring of wild-fires in semi-rural areas.

Among the future work plans of the authors are the further refinement and generalisation of the social media information model harmonised with the NGSIv2 specification, as well as officially proposing it as an extension to the FIWARE-GSMA vocabulary. In addition, the authors plan to further refine and extend the data inference mechanisms. To this end, further experiments realised in the fields will allow for the creation of extended datasets for training more sophisticated inference algorithms. These datasets will be modelled based on the NGSI data model and will be made available to the scientific community for further research. 

## Figures and Tables

**Table 1:** IoT layers, interoperability points and indicative standards.

IoT Layer	Interoperability Point	Standards
Device Layer: Ensures communication between devices on physical device layer and IoT platforms. It also enables transmission of data from devices and reception of actuation commands to targeted devices.	1	LoRa, Lte, Sigfox, 3G, Bluetooth, WiFi, Zigbee
Network Layer: Exposes the raw data generated from IoT devices through different applications level transport protocols based on different paradigms. It also offers interfaces that allow communicating with devices for management or actuation purposes.	2	MQTT, OneM2M, OMALightweightM2M,
Service and Application Layer: Offers the right information to the right application at the right time. These layers are responsible for transforming raw data into information relevant and ready to be consumed by applications, so that smart behaviours are exhibited.	3	OMA NGSI-9 and NGSI-10, ETSI SIG CIM (NGSI-LD), ETSI SAREF, GSMA, OGC
Security and Privacy: Provides the appropriate means and procedures to tackle potential data privacy and security violations. Also, ensures confidentiality, integrity, authentication, authorisation, trust and non-repudiation among the interacting platforms.	4	EU General Data Protection Regulation (GDPR), GSMA IoT Security Guidelines, oneM2M tech spec, TS-0003-V2.12.1 Security Solutions and TR-0008-V2.0.1 Security.

**Table 2:** Introduced NGSIv2 Attributes assigned to “SocialMediaContent” Entity and their mapping to dominant social media platforms.

NGSIv2 Introduced Attributes	Twitter	Facebook
**Message**	Text	Post
**Like**	Favourite	Like
**Reproduce**	Retweet	Share
**Tags**	Tags	Tags

**Table 3:** Sensor measurements range and fire indication.

MESP Sensor	Measurements Range	Fire Indication Thresholds
Infrared	0–1023	<250
Gas	0–1023	>10
Temperature air	0–50 °C	>45 °C
Temperature ground	−55–+125 °C	>50 °C
Relative Humidity	5–100%	<8%
Image classification	0–1	>0.6

**Table 4:** Tweet populations referring to the specific Rafina-Mati fire incident a few days before, during and after the actual incident.

Date	20/7/2018	21/7/2018	22/7/2018	23/7/2018	24/7/2018	25/7/2018	26/7/2018
**Number of Tweets**	0	0	0	1046	4045	2879	2811

**Table 5:** Sample of weather condition measurements for the area where the wild fire was ignited on 23/7/2018 (Source: National Observatory of Athens (http://www.noa.gr/)).

Time	Temperature (Celsius)	Humidity (%)	Wind Speed Max (km/h)
16:30	30.9	35	74
16:40	30.6	34	88.5
16:50	30.5	34	78.9
17:00	30.8	33	83.7

**Table 6:** Input used by the decision making service to estimate the fire danger level and the probability of actual fire occurrence in the conducted experiments regarding the Mati-Rafina fire incident in Greece (23/7/2018).

	Input:			
	Fire Alert Levels Derived from the Four Classification Engines			
Time	MESP (Image)	Weather	Social Media	EFFIS
16:36	INFORMATIONAL	CRITICAL	INFORMATIONAL	HIGH
16:38	INFORMATIONAL	CRITICAL	INFORMATIONAL	HIGH
16:40	INFORMATIONAL	CRITICAL	INFORMATIONAL	HIGH
16:42	MEDIUM	CRITICAL	INFORMATIONAL	HIGH
16:44	HIGH	CRITICAL	INFORMATIONAL	HIGH
16:46	HIGH	CRITICAL	LOW	HIGH
16:48	CRITICAL	CRITICAL	LOW	HIGH
16:50	CRITICAL	CRITICAL	MEDIUM	HIGH
16:52	CRITICAL	CRITICAL	MEDIUM	HIGH
16:54	CRITICAL	CRITICAL	HIGH	HIGH
16:56	CRITICAL	CRITICAL	HIGH	HIGH
16:58	CRITICAL	CRITICAL	CRITICAL	HIGH
17:00	CRITICAL	CRITICAL	CRITICAL	HIGH
17:02	CRITICAL	CRITICAL	CRITICAL	HIGH
17:04	CRITICAL	CRITICAL	CRITICAL	HIGH

## Appendix A

An example of how a Tweet is modeled based on the NGSI information model and the introduced specification presented in Section 4.

json_social_media_content = {
 "id": "urn:ngsi:SMContent:id_112",
 "type": "SocialMediaContent",
  "dateIssued": {
  "type": "DateTime",
  "value": "2018-07-23T16:45:00.00Z"
  },
  "message": {
  "value": "tweet message goes here",
  "type": "Text"
 },
  "reproduced": {
  "value": 10,
  "type": "Integer"
 },
  "liked": {
    "value": 10,
    "type": "Integer"
  },
  "lang": {
  "value": "Text",
  "type": "EN"
 },
  "source": {
  "value": "http://www.twitter.com",
  "type": "URI"
 },
  "hashtags":{
  "value": ["nodejs","python"],
  "type": "List"
 },
  "refUser": {
    "value": " 1036061995651366912 ",
    "type": "Relationship"
  },
  "refPlace":{
    "value": " 01fbe706f872cb32 ",
    "type": "Relationship"
  }
}
json_social_media_user = {
 "id": "urn:ngsi:SMUser:id_112",
 "type": "SocialMediaUser",
  "dateIssued": {
  "type": "DateTime",
  "value": "2018-07-23T16:45:00.00Z"
  },
  "name": {
  "value": "JohnSmith",
  "type": "Text"
 },
  "location": {
  "value": San Francisco, CA",
  "type": "Text"
 },
  "screenName": {
    "value": 10,
    "type": "JohnSmith_account"
  },
  "url": {
  "value": "https://twitter.com/johndoeaccount",
  "type": "URI"
 },
  "description": {
  "value": "This is the original account of JD",
  "type": "Text"
 },
  "profile_image_url_https":{
  "value": "https://pbs.twimg.com/profile_images/1667941687/john_doe_profile_image.jpg",
  "type": "URI"
 },
  "refContent": {
    "value": "urn:ngsi:SMContent:id_112",
    "type": "Relationship"
  },
  "refPlace":{
    "value": "urn:ngsi:PointOfInterest:id_112",
    "type": "Relationship"
  }
}

## References

[1] Liu G., Perez R., Muñoz J.A., Regueira F. (2015). The Internet of Things: Mapping the Value beyond the Hype.

[2] IoT European Platforms Initiative (2018). Advancing IoT Platforms Interoperability.

[3] Serrano M., Barnaghi P., Carrez F., Cousin P., Vermesan O., Friess P. (2015). IoT Semantic Interoperability: Research Challenges, Best Practices, Recommendations and Next Steps.

[4] Bröring A., Schmid S., Schindhelm C.K., Khelil A., Kaebisch S., Kramer D., Le Phuoc D., Mitic J., Anicic D., Teniene E. (2017). Enabling IoT Ecosystems through Platform Interoperability. IEEE Softw..

[5] Ganzha M., Paprzycki M., Pawłowski W., Szmeja P., Wasielewska K. (2017). Semantic interoperability in the Internet of Things: An overview from the INTER-IoT perspective. J. Netw. Comput. Appl..

[6] Jara A.J., Olivieri A., Bocchi Y., Jung M., Kastner W., Skarmeta A. (2014). Semantic Web of Things: An analysis of the application semantics for the IoT moving towards the IoT convergence. Int. J. Web Grid Serv..

[7] Pfisterer D., Romer K., Bimschas D., Kleine O., Mietz R., Truong C., Hasemann H., Kröller A., Pagel M., Hauswirth M. (2011). SPITFIRE: Toward a semantic web of things. IEEE Commun. Mag..

[8] Cisco Systems (2014). Internet of Everything—Connecting the Unconnected. http://share.cisco.com/IoESocialWhitepaper/#/.

[9] Jones N. (2018). Top Strategic IoT Trends and Technologies Through 2023.

[10] Kalatzis N., Routis G., Roussaki I., Papavassiliou S. Enabling data interoperability for federated IoT experimentation infrastructures. Proceedings of the 2nd Global IoT Summit (GIoTS 2018).

[11] Andročec D., Novak M., Oreški D. (2018). Using Semantic Web for Internet of Things Interoperability: A Systematic Review. Int. J. Semant. Web Inf. Syst..

[12] Noura M., Atiquzzaman M., Gaedke M. (2018). Interoperability in Internet of Things: Taxonomies and Open Challenges. Mob. Netw. Appl..

[13] (2012). Next Generation Service Interfaces Architecture (Approved Version 1.0), Open Mobile Alliance, OMA-AD-NGSI-V1_0-20120529-A. http://www.openmobilealliance.org/release/NGSI/V1_0-20120529-A/OMA-AD-NGSI-V1_0-20120529-A.pdf.

[14] NGSI 9/10 Information Model, 2019. http://www.openmobilealliance.org/release/NGSI/.

[15] (2019). FIWARE. https://www.fiware.org/.

[16] (2019). Open & Agile Smart Cities. http://www.oascities.org/open-agile-smart-cities/.

[17] Dolui K., Kiraly C. Towards multi-container deployment on IoT gateways. Proceedings of the IEEE Global Communications Conference (GLOBECOM 2018).

[18] Robert J., Kubler S., Kolbe N., Cerioni A., Emmanuel G., Främling K. (2017). Open IoT ecosystem for enhanced interoperability in smart cities—Example of Métropole de Lyon. Sensors.

[19] Schmid S., Bröring A., Kramer D., Kaebisch S., Zappa A., Lorenz M., Wang Y., Gioppo L. An Architecture for Interoperable IoT Ecosystems. Proceedings of the 2nd International Workshop on Interoperability & Open Source Solutions for the Internet of Things (InterOSS-IoT 2016) at the 6th International Conference on the Internet of Things (IoT 2016).

[20] Ganzha M., Paprzycki M., Pawłowski W., Szmeja P., Wasielewska K. (2018). Towards Semantic Interoperability between Internet of Things Platforms. Integration, Interconnection, and Interoperability of IoT Systems.

[21] Herzog R., Jacoby M., Žarko I.P. (2016). Semantic interoperability in IoT-based automation infrastructures. at-Automatisierungstechnik.

[22] Hovstøa A., Guanb Y., Vásquezb J., Savaghebib M., Guerrerob J., Poveda-Villalónc M., García-Castroc R., Hafenstrom S. Enabling interoperability-as-a-service for connected IoT infrastructures and Smart Objects. Proceedings of the 15th International Conference on Wearable, Micro and Nano Technologies for Personalized Health (pHealth 2018).

[23] (2019). IoT European Large Scale Project. https://european-iot-pilots.eu/.

[24] Varga P., Blomstedt F., Ferreira L.L., Eliasson J., Johansson M., Delsing J., de Soria I.M. (2017). Making system of systems interoperable—The core components of the arrowhead framework. J. Netw. Comput. Appl..

[25] Murdock P., Bassbouss L., Bauer M., Alaya M.B., Bhowmik R., Brett P., Chakraborty R.N., Dadas M., Davies J., Diab W. (2016). Semantic Interoperability for the Web of Things.

[26] Ahmed F., Asif R., Hina S., Muzammil M. (2017). Financial Market Prediction using Google Trends. Int. J. Adv. Comput. Sci. Appl..

[27] Askitas N., Zimmermann K.F. (2009). Google econometrics and unemployment forecasting. Appl. Econ. Q..

[28] O’Connor B., Balasubramanyan R., Routledge B.R., Smith N.A. From tweets to polls: Linking text sentiment to public opinion time series. Proceedings of the 4th AAAI International Conference on Weblogs and Social Media (ICWSM 2010).

[29] Tumasjan A., Sprenger T., Sandner P.G., Welpe I.M. Predicting elections with twitter: What 140 characters reveal about political sentiment. Proceedings of the 4th AAAI International Conference on Weblogs and Social Media (ICWSM 2010).

[30] Sinha S., Dyer C., Gimpel K., Smith N.A. Predicting the NFL Using Twitter. Proceedings of the Machine Learning and Data Mining for Sports Analytics Workshop (ECML/PKDD 2013).

[31] Chauhan A., Kummamuru K., Toshniwal D. (2017). Prediction of places of visit using tweets. Knowl. Inf. Syst. J..

[32] Giannakopoulos O., Kalatzis N., Roussaki I., Papavassiliou S. Gender Recognition Based on Social Networks for Multimedia Production. Proceedings of the 13th IEEE Image, Video, and Multidimensional Signal Processing Workshop (IVMSP 2018).

[33] Kalatzis N., Roussaki I., Matsoukas C., Paraskevopoulos M., Papavassiliou S., Tonoli S. Social Media and Google Trends in Support of Audience Analytics: Methodology and Architecture. Proceedings of the Seventh International Conference on Data Analytics (DATA ANALYTICS 2018).

[34] Kirilenko A.P., Molodtsova T., Stepchenkova S.O. (2015). People as sensors: Mass media and local temperature influence climate change discussion on twitter. Glob. Environ. Chang..

[35] Sakaki T., Okazaki M., Matsuo Y. Earthquake shakes Twitter users: Real-time event detection by social sensors. Proceedings of the 19th International Conference on World Wide Web (WWW).

[36] Ocampo A.J., Chunara R., Brownstein J.S. (2013). Using search queries for malaria surveillance. Thail. Malar. J..

[37] Yang S., Santillana M., Brownstein J.S., Grady J., Richardson S., Kou S.C. (2017). Using electronic health records and Internet search information for accurate influenza forecasting. BMC Infect. Dis..

[38] Boulton C.A., Shotton H., Williams H.T.P. Using Social Media to Detect and Locate Wildfires. Proceedings of the 10th International AAAI Conference on Web and Social Media (ICWSM 2016).

[39] Digital Earth Lab. http://digitalearthlab.jrc.ec.europa.eu/activities/detecting-forest-fires-social-media/57793.

[40] Slavkovikj V., Verstockt S., Van Hoecke S., Van de Walle R. (2014). Review of wildfire detection using social media. Fire Saf. J..

[41] Wang Z., Ye X., Tsou M.H. (2015). Spatial, temporal, and content analysis of Twitter for wildfire hazards. Nat. Hazards.

[42] Abel F., Hauff C., Houben G.J., Stronkman R., Tao K. Twitcident: Fighting fire with information from social web streams. Proceedings of the 21st International ACM Conference on World Wide Web (WWW 2012).

[43] Alkhatib A.A. (2014). A Review on Forest Fire Detection Techniques. Int. J. Distrib. Sens. Netw..

[44] Sharma A.K., Ansari M.F.R., Siddiqui M.F., Baig M.A. (2017). IoT Enabled Forest Fire Detection and online Monitoring System. Int. J. Curr. Trends Eng. Res..

[45] Niranjana R., HemaLatha T. (2018). An autonomous IoT infrastructure for forest fire detection and alerting system. Int. J. Pure Appl. Math..

[46] Toledo-Castro J., Santos-González I., Hernández-Goya C., Caballero-Gil P. Management of Forest Fires Using IoT Devices. Proceedings of the 11th International Conference on Mobile Ubiquitous Computing, Systems, Services and Technologies (UBICOMM 2017).

[47] Basu M.T., Karthik R., Mahitha J., Reddy V.L. (2018). IoT based forest fire detection system. Int. J. Eng. Technol..

[48] Vijayalakshmi S.R., Muruganand S. (2017). Internet of Things technology for fire monitoring system. Int. Res. J. Eng. Technol..

[49] Kubicek H., Cimander R., Scholl H.J. (2011). Organizational Interoperability in E-Government: Lessons from 77 European Good-Practice Cases.

[50] European Commission (2011). European Interoperability Framework for Pan-European Egovernment Services.

[51] D3.3. Opportunities and Barriers in the Present Regulatory Situation for System Development. https://www.iof2020.eu/deliverables/d3.3-opportunities-and-barriers-in-the-present-regulatory-situation-for-system-development-v1.2.pdf.

[52] Standardisation, AIOTI WG03–loT (2018). High Level Architecture (HLA). Release 4.0.

[53] H2020 EU Project SynchroniCity: Delivering an IoT enabled Digital Single Market for Europe and Beyond, Grant Agreement ID: 732240. https://synchronicity-iot.eu.

[54] H2020 Project IoF2020: Internet of Food and Farm 2020, Grant Agreement ID: 731884. https://www.iof2020.eu/.

[55] Bauer M., Kovacs E., Schülke A., Ito N., Criminisi C., Goix L.W., Valla M. The context API in the OMA next generation service interface. Proceedings of the 14th International Conference on Intelligence in Next Generation Networks (ICIN 2010).

[56] Dey A.K. (2001). Understanding and using context. Pers. Ubiquitous Comput..

[57] FIWARE Data Models. https://fiware-datamodels.readthedocs.io.

[58] IoT Big Data Harmonised Data Model, GSM Association, Version 5.0, 19 June 2018. https://www.gsma.com/iot/wp-content/uploads/2018/07/CLP.26-v5.0.pdf.

[59] Context Information Management (CIM), Application Programming Interface (API) ETSI Group Specification CIM 004 V0.0.11 (February 2018). https://docbox.etsi.org/ISG/CIM/Open/ISG_CIM_NGSI-LD_API_Draft_for_public_review.pdf.

[60] FIWARE Cygnus GE, 2019. https://fiware-cygnus.readthedocs.io/en/latest/.

[61] AuthzForce GE, 2019. https://authzforce-ce-fiware.readthedocs.io/en/latest/.

[62] GSMA—IoT Security Guidelines. https://www.gsma.com/iot/iot-security/iot-security-guidelines/.

[63] FIWARE Alert Data Model. https://fiware-datamodels.readthedocs.io/en/latest/Alert/doc/spec/index.html#alert-data-model.

[64] Roussaki I., Kalatzis N., Liampotis N., Kosmides P., Anagnostou M., Doolin K., Jennings E., Bouloudis Y., Xynogalas S. (2012). Context-awareness in wireless and mobile computing revisited to embrace social networking. IEEE Commun. Mag..

[65] San-Miguel-Ayanz J., Durrant T.H., Boca R., Libertà G., Branco A., de Rigo D., Ferrari D., Maianti P., Vivancos T.A., Schulte E. (2017). Forest fires in Europe, Middle East and North Africa 2016.

[66] Aslan Y.E., Korpeoglu I., Ulusoy Ö. (2012). A framework for use of wireless sensor networks in forest fire detection and monitoring. Comput. Environ. Urban Syst..

[67] Vipin V. (2012). Image Processing Based Forest Fire Detection. Int. J. Emerg. Technol. Adv. Eng..

[68] COPERNICUS. http://effis.jrc.ec.europa.eu/applications/data-and-services/.

[69] Kalatzis N., Avgeris M., Dechouniotis D., Papadakis-Vlachopapadopoulos K., Roussaki I., Papavassiliou S. Edge Computing in IoT Ecosystems for UAV-Enabled Early Fire Detection. Proceedings of the 2018 IEEE International Conference on Smart Computing (SMARTCOMP 2018).

[70] Forestry Images. https://www.forestryimages.org/browse/subimages.cfm?sub=740.

[71] Avgeris M., Spatharakis D., Dechouniotis D., Kalatzis N., Roussaki I., Papavassiliou S. (2019). Where there is fire there is SMOKE: A Scalable Edge Computing Framework for Early Fire Detection. Sensors.

[72] Stocks B.J., Lynham T.J., Lawson B.D., Alexander M.E., Wagner C.V., McAlpine R.S., Dube D.E. (1989). The Canadian Forest Fire Danger Rating System: An overview. For. Chron..

[73] Roussaki I. (2018). Deliverable 3.1: Experiments Design, Set-Up and Applications.

[74] 2018 Attica Wildfires. https://en.wikipedia.org/wiki/2018_Attica_wildfires.

[75] Twitter crazypianist30. https://twitter.com/crazypianist30/status/1021392831997521921.

[76] Video Footage Capturing the Moment the Mati-Rafina Fire Broke Out. http://www.ekathimerini.com/231880/article/ekathimerini/news/video-footage-shows-minutes-mati-fire-broke-out.

